# Nanoscale rules governing the organization of glutamate receptors in spine synapses are subunit specific

**DOI:** 10.1038/s41467-022-28504-4

**Published:** 2022-02-17

**Authors:** Martin Hruska, Rachel E. Cain, Matthew B. Dalva

**Affiliations:** 1 Department of Neuroscience, Rockefeller Neuroscience Institute, West Virginia University, 108 Biomedical Road, Morgantown, WV 26506 USA; 2 Department of Neuroscience and Jefferson Synaptic Biology Center, Sidney Kimmel Medical College at Thomas Jefferson University, 233 South 10th Street, Bluemle Life Sciences Building, Room 324, Philadelphia, PA 19107 USA

**Keywords:** Ion channels in the nervous system, Neurotransmitters, Fluorescence imaging, Supramolecular assembly

## Abstract

Heterotetrameric glutamate receptors are essential for the development, function, and plasticity of spine synapses but how they are organized to achieve this is not known. Here we show that the nanoscale organization of glutamate receptors containing specific subunits define distinct subsynaptic features. Glutamate receptors containing GluA2 or GluN1 subunits establish nanomodular elements precisely positioned relative to Synaptotagmin-1 positive presynaptic release sites that scale with spine size. Glutamate receptors containing GluA1 or GluN2B specify features that exhibit flexibility: GluA1-subunit containing AMPARs are found in larger spines, while GluN2B-subunit containing NMDARs are enriched in the smallest spines with neither following a strict modular organization. Given that the precise positioning of distinct classes of glutamate receptors is linked to diverse events including cell death and synaptic plasticity, this unexpectedly robust synaptic nanoarchitecture provides a resilient system, where nanopositioned glutamate receptor heterotetramers define specific subsynaptic regions of individual spine synapses.

## Abstract (web summary)

Glutamate receptors comprise two obligate subunits and two subunits that confer distinct properties and functions to the specific tetramers, which also localize to distinct synaptic spines. Here, the authors use STimulated Emission Depletion nanoscopy (STED) to provide detailed insights into the spatial organization of glutamate receptor types.

## Introduction

All glutamate receptors are heterotetrametric, composed of two obligate subunits (GluA2 or GluN1) and two subunits that help to specify function: GluN2A or GluN2B (NMDARs), or GluA1 or GluA3 (AMPARs)^1–3^. The heterotetrameric subunit composition of individual glutamate receptors tunes the channel’s properties and is linked to specific aspects of synaptic function and plasticity^4^. For instance, GluA1-containing AMPARs are associated with the expression of synaptic plasticity, while GluN2B-containing NMDARs are associated with the induction of plasticity and are thought to be found at more immature and smaller spines^3,5–8^. The differences in the function of glutamate receptor heterotetramers have led to the suggestion that glutamate receptors containing specific subunits are differentially localized in spines of different sizes^5,9–13^. Despite the broad importance of subsynaptic location of glutamate receptor subunits for synaptic function, how glutamate receptor heterotetramers are organized at the nanoscale within spines is unclear.

Adding to the complexity of the synaptic nanoscale architecture, many AMPARs and NMDARs are located outside or distant to synaptic release sites^3,14^. These non-synaptic receptors are more mobile than AMPARs and NMDARs localized to PSDs^15,16^ and are thought to act as a dynamic pool of receptors that could serve as the source of new synaptically localized receptors^3,15,17–19^. While the importance of extrasynaptic and perisynaptic AMPARs and NMDARs for synaptic function has been long recognized, the relationship between the nanoscale organization of both synaptic and non-synaptic glutamate receptors at spine synapses remains controversial.

Synaptic function requires the juxtaposition of pre- and postsynaptic proteins as the precise localization of glutamate receptors relative to synaptic release sites is critical for normal synaptic function^20^. AMPARs and NMDARs have distinct affinities for glutamate, with AMPARs binding glutamate at low affinity and NMDARs binding with higher affinity^1,21^. Electrophysiological and modeling evidence indicates that AMPARs are better coupled to glutamate release sites than NMDARs^13^. Moreover, the location of the heterotetrameric receptor appears important for function, with synaptic receptors signaling to induce events such as long-term potentiation and spine size changes, and non-synaptic receptors’ signaling leading to events such as long-term depression and cell death^14,22–24^. However, the nanoscale relationship between the positioning of AMPARs, NMDARs, and the machinery responsible for release remains unknown.

Here we demonstrate using STimulated Emission Depletion nanoscopy (STED) that the organization of NMDARs and AMPARs reflect the modular structure of pre- and postsynaptic scaffolding proteins, with the number of similar-sized clusters of these proteins scaling with spine size. However, within the modular structure of AMPARs and NMDARs, there is flexibility. Heterotetrameric AMPARs containing the GluA1 subunit and heterotetrameric NMDARs with the GluN2B subunit do not show modules that scale with spine size. Instead, they are preferentially localized to large and small spines, respectively, reflecting their function in synaptic plasticity and development. In addition to a highly ordered post-synaptic nanoarchitecture, the glutamate receptor subtypes are differentially yet precisely positioned relative to the fast calcium sensor SYT1, reflecting differences in how these receptors bind glutamate. Together, our data describe robust organizational principles of essential components of synaptic transmission and plasticity.

## Results

### AMPARs in dendritic spine synapses form nanoscale modules

**Fig. 1:**
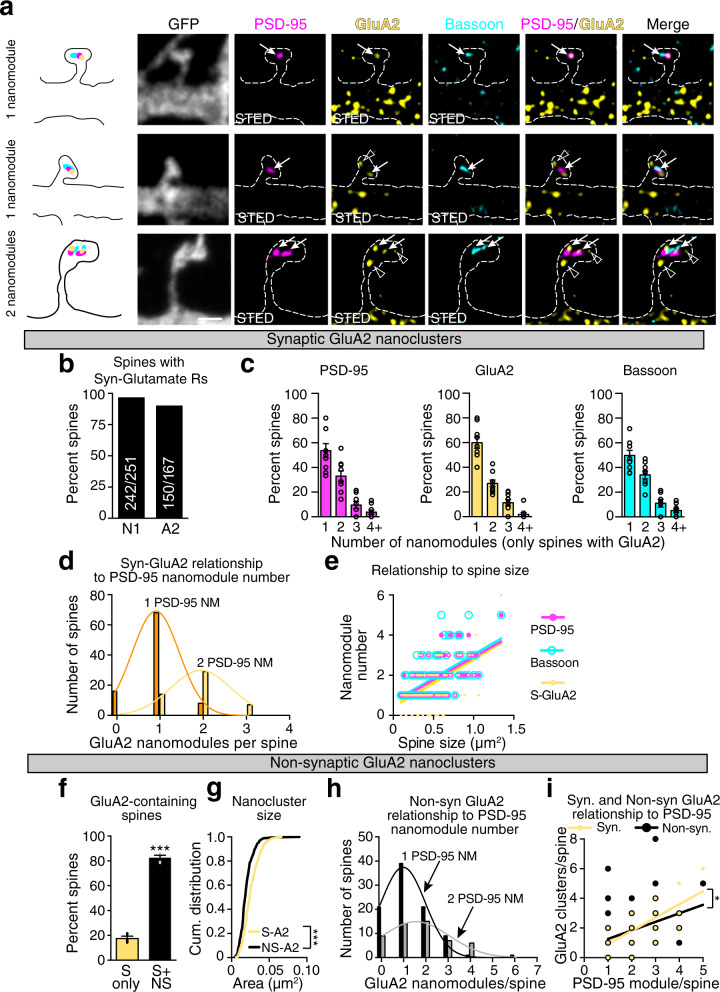
The nanoscale organization of AMPARs in dendritic spine synapses reflects a modular organization of pre- and postsynaptic adaptor molecules. **a** Representative images of single and two-nanomodule spines from DIV21 cortical neurons. Antibodies to PSD-95 (magenta, Atto-425), GluA2 (yellow, Atto-647N), and Bassoon (cyan, AlexaFluor-594) were used to identify the colocalization of AMPARs with synaptic adaptor nanomodules in STED mode (white arrows) in GFP-labeled spines (gray, dotted lines, confocal mode) as well as non-synaptic GluA2 clusters (open arrowheads). Scale bar: 1 µm. **b** Percent of spines containing synaptic NMDARs and AMPARs (numbers of spines are indicated on the graph). **c** Percentage of spines containing single and multiple PSD-95, GluA2, and Bassoon clusters (*n* = 150 spines). **d** Distribution of the number of synaptic GluA2 nanoclusters as a function of the number of PSD-95 nanomodules in a spine. Gaussian fits model distinct distributions of S-GluA2 puncta in one (orange) and two (yellow) PSD-95 nanomodule (PSD-95 NM) spines (*p* < 0.0001 by extra sum of squares, F test). Most spines contain equal numbers of synaptic GluA2 and PSD-95 nanoclusters. **e** Quantification of the relationship between spine size (*n* = 167 spines) and the number of Bassoon (cyan line, *R*^2^ = 0.3537, slope=2.337 ± 0.2459), PSD-95 (magenta line, *R*^2^ = 0.4160, slope = 2.275 ± 0.2099) and GluA2 (yellow line, *R*^2^ = 0.3271, slope = 2.364 ± 0.2639, *p* = 0.9652, one-way ANCOVA) clusters. **f** Percent of spines containing only synaptic (yellow bar, *n* = 30 spines) or both, synaptic and non-synaptic (black bar, *n* = 137 spines) GluA2 nanoclusters (***p < 0.0001, two-tailed Student’s t-test, dots represent averages of data from three individual experiments). **g** Cumulative probability distributions of synaptic and non-synaptic GluA2 nanocluster sizes (***p < 0.0001, two-tailed Kolmogorov–Smirnov (K–S) test). **h** Distribution of the number of non-synaptic GluA2 nanoclusters relative to the number of PSD-95 nanomodules per spine as in (**d**). Distributions of NS-GluA2 puncta in one (black) and two (gray) PSD-95 NMs. The distributions of NS-GluA2 puncta were not related to synaptic PSD-95 puncta number (*p* = 0.1276 by extra sum of squares, F-test). **i** Relationship of the synaptic (slope = 0.8927, *R*^2^ = 0.5828) and non-synaptic (slope = 0.5755, *R*^2^ = 0.3642) GluA2 nanocluster numbers to the number of PSD-95 nanomodules per spine (*p = 0.0143, one-way ANCOVA, *n* = 167 spines). Bars represent the mean ± SEM.

AMPARs mediate the fast component of neurotransmission, and AMPAR abundance is correlated with spine size, determines synaptic strength, and mediates synaptic plasticity^2,17^. Therefore, we asked whether AMPAR nanoclusters in spines follow the modular organization defined by PSD-95 and presynaptic scaffolding proteins in spines^25^. To determine the relationship between glutamate receptors and pre- and post-synaptic proteins that form nanomodules, multi-color STED was chosen because STED enables super-resolution imaging of three colors with minimal chromatic aberration in *XY* or *Z* planes with ~50 nm resolution in XY (Supplementary Figs. 1 and 2a–c), and allows for discrimination of two different fluorophores as close as ~70 nm apart (Supplementary Fig. 2d–j). All STED images were collected using *Z*-resolved STED and deconvolved using the same parameters to improve the signal to noise ratio (compare Fig. 1 and Supplementary Fig. 3). Deconvolution did not lead to significant changes in the distances between objects (Supplementary Fig. 2f, i, and j; see “Methods”) and the size of the puncta examined were on average larger than the resolution limit of our system, measured as the minimal full width at half maximal (FWHM) detected (Supplementary Fig. 2a–c). Thus, STED enables the determination of the distance between different puncta at the nanoscale.

To visualize the heterotetrameric AMPA-type glutamate-receptor channels, we probed for the most commonly incorporated subunit, GluA2^26,27^. EGFP labeled DIV21-25 neurons immunostained with antibodies for endogenous GluA2, PSD-95, and Bassoon were imaged with three-color *Z*-resolved 3D-STED (Fig. 1a and Supplementary Fig. 3). Neuronal morphology was determined in confocal mode, and the organization of synaptic proteins in GFP labeled spines was determined using three-color STED in *XY* and *Z* planes (Fig. 1a and Supplementary Fig. 3), allowing for the organization of synaptic nanostructure to be related to spine size^25^. Consistent with previous results^25^, PSD-95 and Bassoon formed aligned nanomodules that scaled in number with increasing spine size (Fig. 1a–e and Supplementary Fig. 3).

In mature cortical neurons, 89% of spines had GluA2-containing AMPAR heterotetramers (Fig. 1b and Supplementary Fig. 4b). In spines with synaptic GluA2-containing AMPARs, GluA2 subunits localized to 81% of PSD-95 nanomodules (Supplementary Fig. 4a, c). These nanoclusters of GluA2 subunits, adjacent to presynaptic markers and associated with postsynaptic PSD-95, are defined as synaptic nanoclusters (Fig. 1a, arrows and Supplementary Fig. 3, arrowheads). Non-synaptic GluA2 nanoclusters were also found in many spines (Fig. 1a, open arrowheads). To determine whether GluA2 synaptic nanoclusters form modular assemblies that scale with spine size, we determined the relationship between the number of GluA2 and PSD-95 nanoclusters in spines of different sizes. The number of GluA2 nanoclusters increased as a function of the number of PSD-95 clusters (Fig. 1d). Additionally, the number of synaptic GluA2 nanoclusters scaled linearly with spine size (Fig. 1e). The relationship of synaptic GluA2 nanocluster number to spine size was not significantly different from the scaling seen with PSD-95 and Bassoon nanomodules. These data indicate that synaptic AMPARs form nanoclusters in the same modular fashion as PSD-95.

**Fig. 2:**
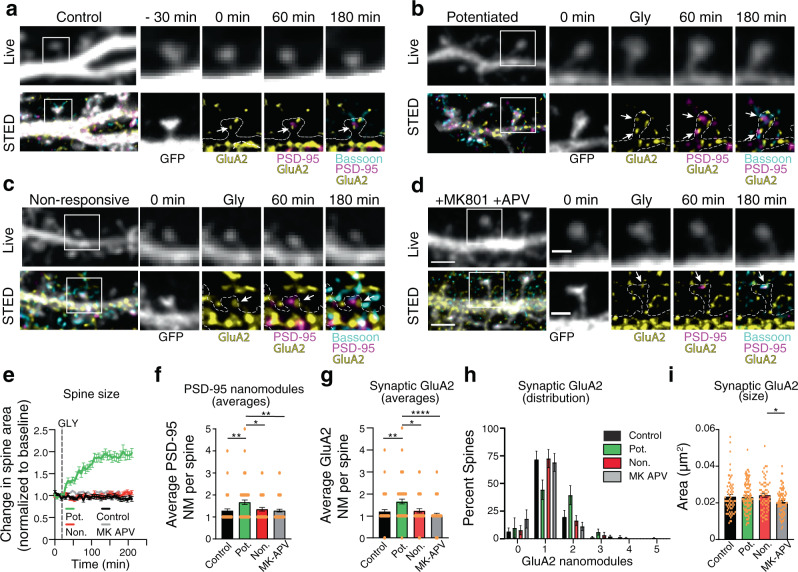
NMDAR dependent structural plasticity is linked to increased number of synaptic GluA2 nanomodules. **a**–**d** Representative three-hour time-lapse images (top panels, confocal resolution) and post hoc STED images (bottom panels) of the same dendritic spines (white squares) of GFP-transfected DIV21-25 cortical neurons. Cells were retrospectively stained and imaged for cell morphology in confocal (gray, outline) and three-color STED of endogenous GluA2 (yellow, Atto-647N), PSD95 (magenta, Atto-425), and Bassoon (cyan, AlexaFluor-594). Arrows represent triple-colocalized GluA2/PSD95/Bassoon clusters. **e** Quantification of change in spine area, normalized to baseline area, over three hours following treatment with glycine (3 min, 200 µM). Potentiated spines were defined by a sustained increase in spine area of >10% over baseline (Pot., green traces, *n* = 64 spines), non-responsive spines were defined as those that did not increase in spine area (Non., red traces, *n* = 51 spines). Spine enlargement was blocked by treatment with 50uM APV and 10 μM MK-801 (gray traces, *n* = 79 spines). Control spines were not subjected to glycine treatment (black traces, *n* = 56 spines). Graph represents mean ± SEM. **f**, **g** Quantification of average number of PSD-95 (**f**) (**p = 0.002, one-way ANOVA with Tukey’s post hoc) and synaptic GluA2 (**g**) (****p < 0.0001, one-way ANOVA with Tukey’s post hoc) modules per spine in the indicated conditions following retrospective STED imaging. **h** Distributions of spines with single and multiple synaptic GluA2 modules in the indicated conditions, binned on the basis of the number of synaptic GluA2 clusters they contained. Conditions and spine numbers in (**f**–**h**) as in (**e**). **i** Quantification of the average area of individual synaptic GluA2 clusters in the indicated conditions (control: *n* = 67 clusters; Pot: *n* = 106 clusters; Non.: *n* = 63 clusters; MK-APV: *n* = 83 clusters; *p = 0.041, one-way ANOVA, Tukey’s posthoc). Bar graphs represent mean ± SEM, with the number of individual spines or clusters represented by dots. *p < 0.05, **p < 0.005, ***p < 0.0001. Scale bar for Live panel in **d**: 2 µm, applicable to Live panels in (**a**–**d**). Scale bar for STED panel in **d**: 2 µm, applicable to STED panels in (**a**–**d**). Scale bar for Live inset in **d**: 1 µm, applicable to Live insets in (**a**–**d**). Scale bar for STED inset in **d**: 1 µm, applicable to STED insets in (**a**–**d**).

The number, but not size, of PSD-95 nanomodules increases with plasticity-induced spine enlargement^25^. Therefore, we asked whether GluA2 nanoclusters might undergo similar changes in response to chemical LTP^18,25,28,29^. Spine enlargement was induced by application of glycine (3 min, 200 µM) to DIV21-25 cortical neurons transfected with GFP. Neurons were imaged with confocal spinning disk or confocal Leica SP8 every 6 min for 3 h post glycine application^25^. After live imaging, neurons were fixed and stained for endogenous GluA2, PSD-95, and Bassoon (Fig. 2a–d).

Glycine application results in significant increase in the size of 56% of spines (potentiated), with the remaining non-responsive spines having no lasting changes in spine size (Fig. 2b, e). Retrospective three-color STED analysis visualized the nanoscale organization of GluA2, PSD-95, and Bassoon of individual spines that were imaged live. Potentiated spines had significantly more nanoclusters of synaptic GluA2 and PSD-95 nanomodules than control, non-responsive or MK-801/APV treated spines (Fig. 2f–h). Consistent with the model that GluA2 forms nanomodules there was no difference in the size of GluA2 nanoclusters following induction of structural plasticity (Fig. 2i). These results suggest that GluA2 forms nanomodules that scale in number with changes in spine size similar to the modular organization of other synaptic proteins^25^.

AMPARs dynamically associate with synapses and undergo constitutive recycling and delivery to synaptic sites to maintain synaptic strength^17^. Consistent with this model, the majority of spines containing synaptic AMPARs (>80%) had non-synaptic nanoclusters of GluA2 subunit-containing AMPAR heterotetramers (Fig. 1f). Non-synaptic GluA2 nanoclusters were significantly smaller than synaptic GluA2 clusters (Fig. 1g, Avg. S-GluA2 size = 0.025 µm^2^, Avg. NS-GluA2 size = 0.019 µm^2^, *p* < 0.0001, K–S test). Unlike synaptic GluA2 nanoclusters, the non-synaptic clusters were found in higher numbers and were less well related to PSD-95 nanomodule numbers (Fig. 1h, i). These data suggest that, unlike synaptic GluA2 heterotetramer nanoclusters, non-synaptic clusters are not modular.

### Organization of AMPARs at spine synapses follows subunit-specific rules

**Fig. 3:**
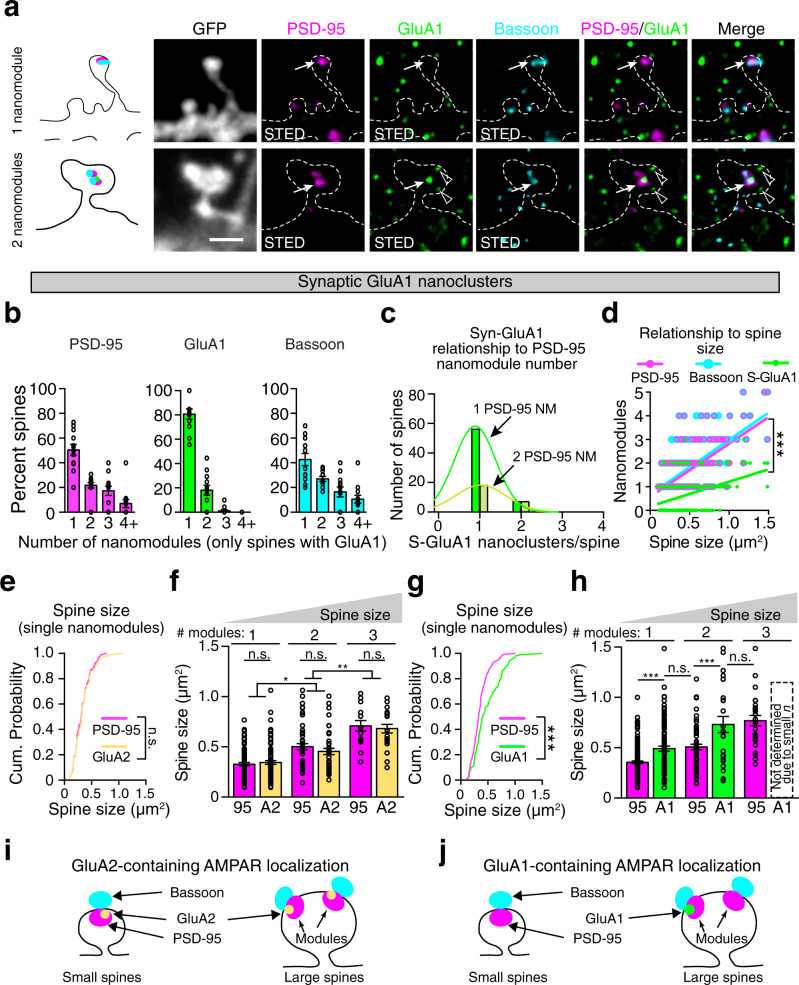
GluA1 containing AMPARs are found in large spines and are organized in a non-modular manner. **a** Images of single and multi-nanomodule spines from DIV21 GFP transfected cortical neurons stained for PSD-95 (magenta, Atto-425), GluA1 (green, Atto-647N), and Bassoon (cyan, AlexaFluor-594). Colocalization indicated by white arrows; arrowheads indicate GluA1 clusters alone. Scale bar: 1 µm. **b** Percentage of spines containing single and multiple PSD-95, GluA1, and Bassoon clusters (*n* = 128 spines). **c** Distribution of the number of synaptic GluA1 nanoclusters as a function of the number of PSD-95 nanomodules. Gaussian fits model two overlapping distributions of S-GluA1 puncta in one (green) and two (yellow) PSD-95 NM spines (*p* < 0.0001 by extra sum of squares, F test), indicating that most spines contain only one synaptic GluA1 puncta, regardless of the number of PSD-95 puncta. **d** Quantification of the relationship between spine size (*n* = 240 spines) and the number of Bassoon (cyan line, *R*^2^ = 0.4076, slope = 2.306 ± 0.1802), PSD-95 (magenta line, *R*^2^ = 0.4330, slope = 2.246 ± 0.1666) and GluA1 (yellow line, *R*^2^ = 0.1515, slope = 1.040 ± 0.1596, ***p < 0.0001, one-way ANCOVA) nanopuncta. Comparison of PSD-95 and GluA2’s relationship to spine size in spines with (**e**) single (PSD-95, *n* = 92 spines, GluA2, *n* = 90 spines, *p* > 0.9999, two-tailed K–S test) and **f** multiple PSD-95 (*n* = 52 two-clustered spines, *n* = 15 three-clustered spines) and GluA2 (*n* = 42 two-clustered spines, *n* = 21 three-clustered spines, *p < 0.018, **p < 0.034, one-way ANOVA, Tukey’s post hoc) clusters. Comparison of PSD-95 and GluA1’s relationship to spine size in spines with (**g**) single (PSD-95, *n* = 134 spines, GluA1, *n* = 100 spines, ***p = 0.007, two-tailed K–S test,) and **h** multiple PSD-95 (*n* = 61 two-clustered spines, *n* = 26 three-clustered spines) and GluA1 (*n* = 25 two-clustered spines, ***p < 0.006, one-way ANOVA, Tukey’s post hoc) nanomodules. Model of GluA2 (**i**) and GluA1 (**j**) localization to small and large spines. Bars represent the mean ± SEM. *P* values for comparisons in **f**, **h** are provided in the source data.

GluA1 subunit-containing AMPAR heterotetramers can be incorporated into synapses with the expression of structural plasticity^5,30,31^. The relationship between spine size, plasticity, and GluA1-containing AMPARs suggests that GluA1-containing receptors represent a subset of AMPARs that do not follow a modular organization, allowing for more flexibility in the organization of spine synapses. The pattern of GluA1-containing AMPARs at spine synapses was visualized using antibodies against endogenous GluA1, and synaptic sites were determined by staining for endogenous PSD-95 and Bassoon (Fig. 3a and Supplementary Fig. 5). The majority of GluA1 subunits form heterotetramers with GluA2^2^, therefore we expect that most GluA1 puncta would colocalize with GluA2. Consistent with this model, co-staining with antibodies against GluA1 and GluA2 subunits indicates that the majority of GluA1 nanoclusters colocalize with GluA2 clusters (90%, Supplementary Fig. 4a–d). As expected, since GluA2 can tetramerize with both GluA1 and other AMPAR subunits (e.g., GluA3)^27^, only ~50% of GluA2 subunit clusters at synapses colocalize with GluA1 (Supplementary Fig. 4d). These data suggest that AMPAR complexes containing GluA1 subunits and those lacking GluA1 subunits might not share the same nanoscale organization.

Consistent with the idea that AMPARs lacking GluA1 and those containing GluA1 behave differently at the nanoscale, only 53% of spines have GluA1-containing AMPAR puncta (Supplementary Fig. 4b). Within spines at sites marked by nanoclusters of PSD-95 and Bassoon, GluA2 colocalized with 81% of PSD-95 nanomodules (Supplementary Fig. 4a–c, GluA2 (A2) = 40%, GluA1 and GluA2 (A1 + A2) = 41%, Total GluA2 = 81% of PSD-95 nanomodules). In contrast, GluA1 immunostaining was restricted to a subset of PSD-95 nanomodules (Supplementary Fig. 4a–c, GluA1 (A1) = 5%, GluA1 and GluA2 (A1 + A2) = 41%, Total GluA1 = 46% of PSD-95 nanomodules). Of the 53% of spines that contained GluA1 immunostaining at synapses, 80% had only a single GluA1 nanocluster (Fig. 3a, b and Supplementary Fig. 5). In line with this skewed distribution, GluA1 cluster numbers did not scale as a function of PSD-95 nanomodule numbers. In spines with either one or two PSD-95 nanomodules, most spines contained only a single GluA1 nanocluster (Fig. 3c). Moreover, GluA1 cluster numbers scaled significantly less with spine size than PSD-95 and Bassoon, or GluA2 (S-GluA2 (Fig. 1e) vs. S-GluA1 (Fig. 3d), *p* < 0.0001, one-way ANCOVA). Together these data suggest that GluA1-containing AMPARs might not respect the modular organization of adapter proteins.

GluA1 is linked to the expression of plasticity, suggesting that GluA1-containing AMPARs might be preferentially localized to larger spines while GluA2 heterotetramers might be more ubiquitously distributed^5^. To test whether GluA1 and GluA2 subunits may follow different organizational logic at spines, the distribution of spine sizes with single GluA2 or GluA1 clusters was compared to the distribution of spines with single PSD-95 clusters (Fig. 3e–h). Consistent with the model that GluA2 subunits form nanomodules that scale with spine size as a function of PSD-95 nanomodule numbers (Fig. 1d), neither the distribution of sizes nor the average size of GluA2-containing spines was different from spines containing the same number of PSD-95 nanomodules (Fig. 3e, f). In contrast, both the cumulative distribution of spine sizes and the average size of spines containing one GluA1 cluster were significantly larger than spines containing a single PSD-95 nanomodule (Fig. 3g, h). Indeed, the mean size of spines containing a single GluA1 nanocluster was similar to the size of spines with two PSD-95 nanomodules. These data are consistent with a model where GluA1-containing AMPARs are found preferentially in larger spines, while GluA2 has a distribution similar to PSD-95. Thus, overall GluA2-containing AMPARs respect the modular organization of PSD-95, while AMPARs with GluA1-subunits are preferentially localized to larger spines (Fig. 3i, j), consistent with the link between GluA1-containing AMPARs and synaptic plasticity^2,6,17^.

**Fig. 4:**
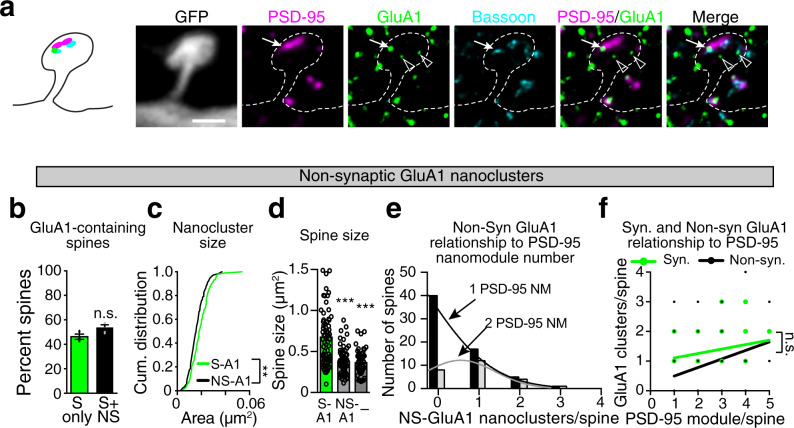
Non-synaptic GluA1 clusters localize to small and large spines in a non-modular manner. **a** Image of a two-nanomodule spine from DIV21 GFP transfected cortical neuron stained for PSD-95 (magenta, Atto-425), GluA1 (green, Atto-647N), and Bassoon (cyan, AlexaFluor-594). White arrows indicate synaptic GluA1 nanoclusters; arrowheads indicate non-synaptic GluA1. Scale bar: 1 µm. **b** Percent of spines containing only synaptic (green bar, *n* = 112 spines) or both, synaptic and non-synaptic (black bar, *n* = 125 spines) GluA1 nanoclusters (*p* = 0.07, two-tailed Student’s t-test, dots represent averages of data from three individual experiments). **c** Cumulative probability distributions of synaptic and non-synaptic GluA2 nanocluster sizes (**p = 0.004, two-tailed K–S test). **d** Average spine size of spines containing both non-synaptic and synaptic GluA1 nanoclusters (S-A1, *n* = 65 spines), only non-synaptic GluA1 (NS-A1, *n* = 60 spines), or lacking GluA1 containing AMPARs (-, *n* = 50 spines, ***p < 0.0001, one-way ANOVA with Tukey’s post hoc). **e** Distribution of the number of non-synaptic GluA1 nanoclusters relative to the number of PSD-95 nanomodules per spine (One PSD-95 NM spines black, Two PSD-95 NM gray). Gaussian fits were used to model non-synaptic GluA1 nanocluster distribution (*p* < 0.0001 by extra sum of squares, F test). The number of NS-GluA1 puncta was not related to PSD-95 puncta number. **f** Relationship of the synaptic (slope = 0.1524, *R*^2^ = 0.1204) and non-synaptic (slope = 0.2878, *R*^2^ = 0.1164) GluA1 nanocluster numbers to the number of PSD-95 nanomodules per spine (*p* = 0.086, one-way ANCOVA, *n* = 127 spines, only spines containing synaptic GluA1 were included). Bars represent the mean ± SEM.

Non-synaptic GluA1 nanoclusters (Fig. 4a, b; open arrowheads in a) were similar to non-synaptic GluA2 clusters (Fig. 1g) as the average size of non-synaptic GluA1 nanoclusters was smaller than synaptic GluA1 clusters (Fig. 4c, Avg. S-GluA1 size = 0.019 µm^2^, Avg. NS-GluA1 size = 0.016 µm^2^, *p* = 0.0002, two-tailed K–S test). However, the proportion of all spines containing non-synaptic GluA1 clusters was significantly lower than that observed for non-synaptic GluA2 clusters (Supplementary Fig. 6a, GluA1 = 54%, GluA2 = 82%, *p* = 0.0019, one-way ANOVA). Of spines with non-synaptic GluA1, spines that contained both non-synaptic and synaptic GluA1 nanoclusters were significantly larger than spines with only non-synaptic GluA1 nanoclusters (Fig. 4d). These data are consistent with a model where non-synaptic GluA1 is positioned in smaller spines to be delivered to synaptic sites upon plasticity inducing stimuli^17,31,32^. Spines frequently contained either no non-synaptic GluA1 or different numbers of non-synaptic GluA1 puncta than would be expected from the number of PSD-95 puncta in the same spine. Thus, the number of non-synaptic GluA1-containing AMPAR nanoclusters found in spines was not well-related to PSD-95 nanomodule number or spine size (Fig. 4a–e, f; empty arrowheads in a). Indeed, of spines containing synaptic GluA1 nanoclusters (GluA1 puncta that colocalized with both PSD-95 and Bassoon) only 50% had non-synaptic GluA1 nanoclusters (Fig. 4b). These data indicate that non-synaptic GluA1 puncta are neither modular nor form structures that appear linked to the modular synaptic scaffold as defined by PSD-95.

### Nanoscale organization of NMDARs in dendritic spine synapses

**Fig. 5:**
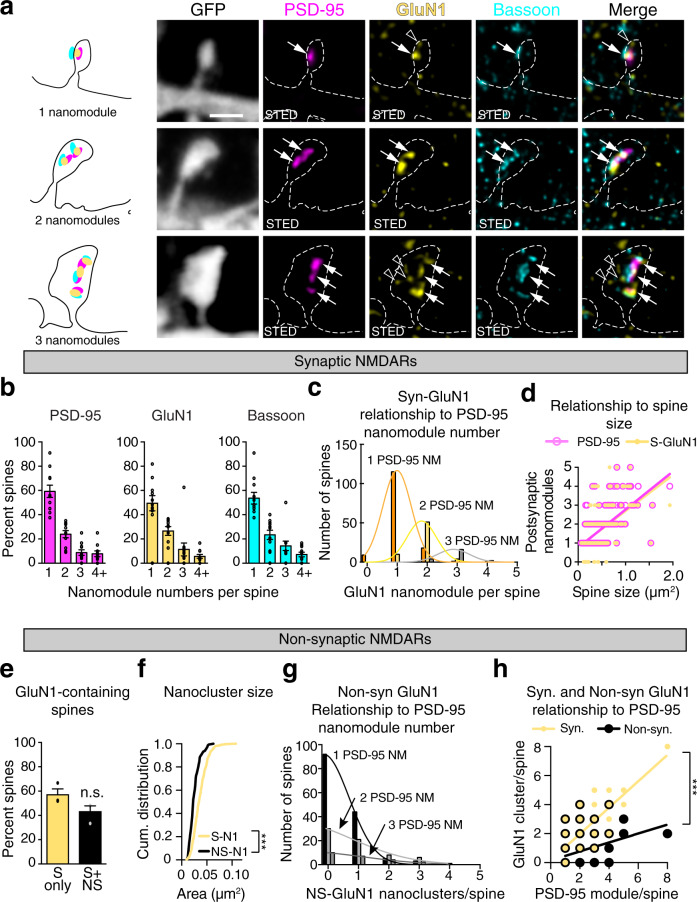
Nanoscale organization of NMDARs in dendritic spine synapses reflects the modular organization of pre- and postsynaptic adaptor molecules. **a** Representative images of single, two, and three nanomodule spines from DIV21 cortical neurons. GFP (gray, dotted lines) was used to visualize spine morphology in confocal mode. Antibodies to PSD-95 (magenta, Atto-425), GluN1 (yellow, Atto-647N), and Bassoon (cyan, AlexaFluor-594) were used to identify adapter protein nanomodules and NMDARs in STED mode (arrows). Open arrowheads indicate non-synaptic GluN1 nanoclusters. Scale bar: 1 µm. **b** Percentage of spines containing single and multiple PSD-95, GluN1, and Bassoon clusters (*n* = 251 spines). **c** Distribution of the number of synaptic GluN1 nanoclusters as a function of PSD-95 nanomodule numbers per spine. Gaussian fits model distinct distributions of S-GluN1 puncta in one (orange), two (yellow), and three (gray) PSD-95 NM spines. The three distributions were non-overlapping and could not be fit with a single distribution (*p* < 0.0001 by extra sum of squares, F test). **d** The number of PSD-95 and synaptic GluN1 nanomodules scales linearly with spine size indistinguishable from each other (PSD-95-magenta line, *R*^2^ = 0.4138, slope = 2.015 ± 0.1523; GluN1-yellow line, *R*^2^ = 0.3326, slope = 1.902 ± 0.1711, *p* = 0.8364, one-way ANCOVA, *n* = 250 spines). **e** Percent of spines containing only synaptic (yellow bar, *n* = 137 spines) or both, synaptic and non-synaptic (black bar, *n* = 114 spines) GluN1 nanoclusters (*p* = 0.6806, two-tailed Student’s t-test, dots represent averages of data from three individual experiments). **f** Cumulative probability distributions of synaptic and non-synaptic GluN1 nanocluster sizes (***p < 0.0001, two-tailed K–S test). **g** Distribution of the number of non-synaptic GluN1 nanoclusters relative to the number of PSD-95 nanomodules per spine as in (**c**). Distributions were overlapping with the same means for both data sets (*p* = 0.3990 by extra sum of squares F-test). Gaussian fits model distinct distributions of NS-GluN1 puncta in one (black), two (light-gray), and three (dark-gray) PSD-95 NM spines. The number of NS-GluN1 puncta was not related to PSD-95 puncta number. **h** Relationship of synaptic (slope = 0.9076, *R*^2^ = 0.7346) and non-synaptic (slope = 0.3076, *R*^2^ = 0.3076) GluN1 nanocluster numbers to the number of PSD-95 nanomodules per spine (*p* < 0.0001, one-way ANCOVA, *n* = 249 spines). Bars represent the mean ± SEM.

NMDARs are thought to be found in most spines, even in some spines that do not contain AMPARs^3^. At spines, NMDARs mediate the induction of synaptic plasticity and NMDAR activation induces increases in the number of pre- and postsynaptic adapter protein nanomodules that underlie structural plasticity^25^. Therefore, we examined whether NMDAR organization might correspond to the modular organization of PSD-95 in spines of different sizes. The nanoscale organization of the NMDAR in dendritic spines was visualized in EGFP-filled DIV21-25 neurons by immunostaining with antibodies against the endogenous GluN1 subunit of the NMDAR along with antibodies against endogenous PSD-95 and Bassoon (Fig. 5a and Supplementary Fig. 7). This approach enabled us to identify synaptic NMDARs which colocalized with both PSD-95 and Bassoon.

In cortical neurons, nearly all spines (96%) contained synaptically-localized GluN1 subunits, indicating the presence of NMDAR heterotetramers at most synapses (Fig. 1b and Supplementary Fig. 4b). Taken together with the GluA2 subunit distribution in spines (Fig. 1b and Supplementary Fig. 4b) and PSD-95 nanomodules (Supplementary Fig. 4c), approximately 7% of spines and 14% of PSD-95 nanomodules contain only NMDARs. These data are consistent with the observation that many spines lack AMPARs and might be ‘silent’ at resting membrane potentials^7,8,27^.

The NMDAR receptor complex interacts with PSD-95^33^. Therefore, we asked if the distribution of GluN1 puncta in spines resembled that of PSD-95 and Bassoon. The fraction of spines with one, two, three, or four synaptic GluN1 puncta was similar to the number of PSD-95 and Bassoon nanomodules, suggesting that GluN1 might also form modules (Fig. 5a, b and Supplementary Fig. 7). Consistent with this model, the number of synaptic GluN1 nanoclusters varied with the number of PSD-95 nanomodules (Fig. 5c). Moreover, the number of synaptic GluN1 clusters scaled linearly with spine size (Fig. 5d). These data suggest that GluN1 forms synaptic nanomodules that scale in number as spine size increases.

Similar to GluA1, approximately 40% of spines contain GluN1 clusters that do not colocalize with synaptic markers (Fig. 5a–e; open arrowheads in 3a, 4a, and Supplementary Fig. 6a). These non-synaptic GluN1 nanoclusters were significantly smaller in size than synaptic GluN1 nanoclusters (Fig. 5f, Avg. S-GluN1 size = 0.036 µm^2^, Avg. NS-GluN1 size = 0.026 µm^2^, *p* < 0.0001, two-tailed K–S). The number of non-synaptic nanoclusters was poorly linked to PSD-95 nanomodule numbers (Fig. 5g, h), with no clear relationship to spine size (Supplementary Fig. 6b). Together these data indicate that in contrast to non-synaptic GluN1 nanoclusters, synaptic GluN1 subunits scale in number as spine size increases, suggesting that they are organized in a modular fashion.

### Subunit-specific organization of NMDARs within spine synapses

**Fig. 6:**
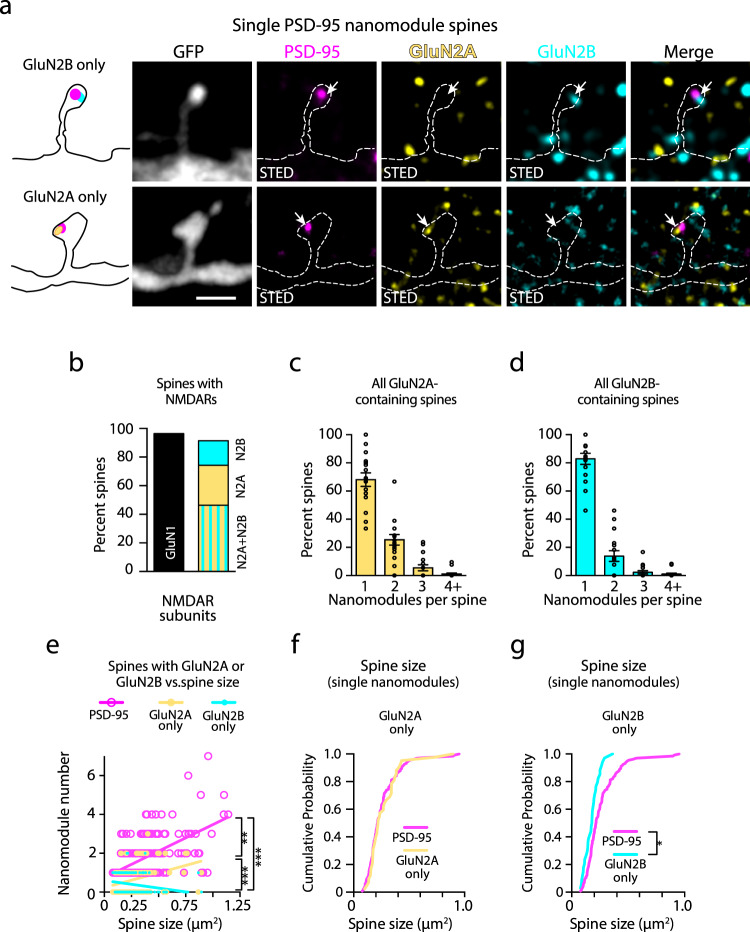
The nanoscale organization of GluN2A and GluN2B NMDAR subtypes in dendritic spine synapses with single PSD-95 nanomodules. **a** Three-color STED images of a single PSD-95 nanomodule (magenta, Atto-425) spine with colocalized GluN2A (yellow, AlexaFluor-594) and GluN2B (cyan, Atto-647N) clusters. Arrows indicate PSD-95 nanomodules containing only GluN2A or only GluN2B. Scale bars: 1 µm. **b** Percent of spines containing NMDAR subunits (GluN1 = 242/251 spines, GluN2A + GluN2B = 112/239 spines, GluN2A only = 67/239 spines, GluN2B only = 40/239 spines). **c**, **d** Percent of spines with single and multiple GluN2A (**c** *n* = 175 spines) and GluN2B (**d** *n* = 154 spines) cluster numbers per spines. **e** Plots of the relationship between spine size and the number of PSD-95 (magenta, *R*^2^ = 0.3391, slope = 2.728 ± 0.2473), GluN2A only (yellow, *n* = 132 spines, *R*^2^ = 0.1179, slope = 1.522 ± 0.3652) and GluN2B only nanomodules (cyan, *n* = 127 spines, *R*^2^ = 0.0508, slope = −0.7803 ± 0.3015; ***p < 0.0001, **p = 0.0138, one-way ANCOVA). **f**, **g** Cumulative probability distributions as a function of spine size in spines with a single PSD-95 nanomodule (magenta, *n* = 133 spines) vs. spines with a single GluN2A cluster only (**f** yellow, *n* = 42 spines, *p* = 0.9466, two-tailed K–S test) or GluN2B cluster only (**g** cyan, *n* = 34 spines, *p = 0.04, two-tailed K–S test). Bars represent the mean ± SEM.

**Fig. 7:**
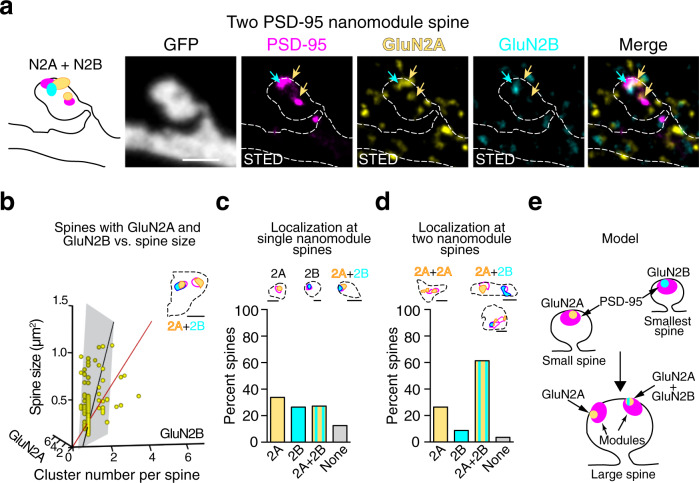
The nanoscale organization of GluN2A and GluN2B NMDAR subtypes in dendritics spines with multiple PSD-95 nanomodules. **a** Three-color STED image of a two PSD-95 nanomodule (magenta, Atto-425) spine with colocalized GluN2A (yellow, AlexaFluor-594) and GluN2B (cyan, Atto-647N) clusters. Arrows indicate PSD-95 nanomodules containing GluN2B (cyan arrow) and/or GluN2A (yellow arrow). Scale bars: 1 µm. **b** A multivariable linear regression of spine size as a function of the number of GluN2A (*p* = 6.34 × 10^−9^, F-statistics) and GluN2B (*p* = 0.28, F-statistics) clusters in spines containing both GluN2A and GluN2B (*n* = 110 spines). Regression plane (gray square) and the best fit line (black) were determined by the coefficients for GluN2A (0.417 ± 0.06), GluN2B (0.26 ± 0.23), and the intercept (0.53 ± 0.11). The line of unit slope is shown in red. Inset: An example of the most commonly occurring localizations of GluN2A clusters (yellow) and GluN2B clusters (cyan) relative to PSD-95 nanomodules (magenta) in spines containing both types of GluN2 clusters. A binary image is shown. Spine head area is outlined by a dotted line. Scale bar: 500 nm. Distribution of NMDAR subunits in spines with **c** single (*n* = 136 spines) and **d** two (*n* = 57 spines) PSD-95 nanomodules. Insets: Binary image examples of GluN2A (yellow) and GluN2B (cyan) nanocluster localizations in spines with **c** one and **d** two PSD-95 nanomodules (magenta). Spine head area is outline by a dotted line. Scale bar: 500 nm. **e** Model of GluN2A and GluN2B cluster distribution in small and large spines. Bars represent the mean ± SEM.

The synaptic content of GluN2A and GluN2B NMDAR subunits changes the ability of a synapse to undergo structural plasticity, suggesting that the specific ratio of GluN2A to GluN2B in NMDAR heterotetramers may tune synaptic function^1,9,10^. Therefore, we examined the distribution of endogenous GluN2A and GluN2B in spines relative to PSD-95 nanomodules (Figs. 6a, 7a and Supplementary Fig. 8). Analysis of the number of GluN2 clusters in spines suggests a more flexible relationship to spine size than seen for the obligatory GluN1 subunit, likely reflecting distinct functional roles of GluN2 subunits in developing (GluN2B) and mature (GluN2A) spines^9,34,35^. Consistent with this more flexible organization, the pattern of GluN2 subunits in spines varied. An array of different combinations of GluN2 subunit distributions were found: from spines with only GluN2A or only GluN2B subunits (Fig. 6a and Supplementary Fig. 8a–d), to spines with both GluN2A and GluN2B subunits either in separate nanoclusters or in the same nanocluster (Fig. 7a, cyan, and yellow arrows, and Supplementary Fig. 8e, f, also see Fig. 7b–d binary image insets). These observations suggest that GluN2 nanoclusters might not follow a strict modular organization with spine size increases.

Nearly all spines (96%) contained the obligatory GluN1 subunit at synapses (Fig. 1b and Supplementary Fig. 4b), but the distribution of endogenous GluN2 subunits was heterogeneous. Approximately 25% of spines contained only GluN2A, 20% contained only GluN2B, while 45% contained both GluN2A and GluN2B (Fig. 6b). To define the relationship between NMDAR subtypes and spine size, we determined the distribution of synaptic GluN2 puncta numbers in individual spine synapses. Similar to GluN1 and PSD-95, we found that approximately 65% of spines that contained GluN2A puncta had a single GluN2A cluster, 25% had two, and less than 5% had three or more GluN2A clusters (Fig. 6c). In contrast, 83% of spines with GluN2B puncta contained a single GluN2B cluster (Fig. 6d). These data suggest that nanoscale clusters of NMDARs that contain GluN2B subunits might not scale with spine size.

Analysis of spines with only GluN2A nanoclusters revealed that the number of GluN2A clusters increased with spine size (Fig. 6e, *p* < 0.0001, Pearson’s correlation). However, consistent with a more flexible relationship to spine size for the GluN2 subunits, GluN2A-containing NMDARs scaled significantly less well with spine size than PSD-95 (*p* = 0.0138, one-way ANCOVA), likely due to many larger spines that contained both GluN2A and GluN2B subunits (Fig. 7d). In contrast, analysis of the relationship between spine size and GluN2B nanocluster numbers in spines containing only GluN2B-containing NMDARs revealed a negative correlation with spine size (Fig. 6e, *p* = 0.01, Pearson’s correlation). Moreover, the distribution of spine sizes indicates that spines containing single puncta of GluN2B are significantly smaller than those that contain single puncta of PSD-95 or GluN2A (Fig. 6f, g, *p* = 0.04, two-tailed K–S test). These data suggest that as spines become larger, they are less likely to contain only GluN2B-containing NMDAR heterotetramers.

In spines that contained both GluN2A and GluN2B nanoclusters, the total number of clusters was positively correlated and increased with spine size (Fig. 7b). Consistent with the model that GluN2B cluster numbers do not increase with spine size, most spines contained only a single GluN2B nanocluster (Fig. 7b inset), skewing the relationship toward GluN2A (GluN2A: *p* < 0.0001, F-statistics, GluN2B: *p* = 0.28, F-statistics). These data suggest that when present in larger spines, GluN2B-containing NMDARs tend to be localized to a single nanocluster, while the number of GluN2A-containing NMDAR nanoclusters increases with spine size.

We next examined whether GluN2 subunits formed separate clusters or were found together at synapses. In smaller spines with single nanomodules of PSD-95, approximately equal proportions of spines (25–35%) had clusters of GluN2A alone, GluN2B alone, or both GluN2A and GluN2B (Fig. 7c and Supplementary Fig. 9a–c). These data suggest that at single PSD-95 nanomodules, GluN2A and GluN2B clusters can co-localize. Consistent with these data, 60% of the larger spines with two PSD-95 nanomodules contained both GluN2A and GluN2B while a minority (8.7%) of the two nanomodule spines contain GluN2B only (Fig. 7d). Analysis of individual PSD-95 nanomodules in these larger spines indicates that a higher proportion of nanomodules colocalize with both GluN2A and GluN2B than in smaller single nanomodule spines (37% vs. 25%, Supplementary Fig. 9c, d). To determine how GluN2 subunits localize within the context of the modular organization of the synapse, we examined PSD-95 nanomodules that co-localize with both GluN2 subunits in spines. At these spine-localized PSD-95 nanomodules, the majority of GluN2A and GluN2B puncta overlap (86%). Interestingly, the overlap was not complete, with an average area of colocalization of only 29.7% (Supplementary Fig. 9e, f). Together with data that GluN2A and GluN2B subunits segregate within the synapse^36^, our data suggest that GluN2 subunits follow subunit-specific rules that govern their organization in spines. GluN2B subunits are found in the smallest spines, while GluN2A-containing NMDARs form nanomodules that scale with spine size. These data are consistent with the distinct functions of GluN2B containing NMDARs during development and the induction of plasticity, and GluN2A NMDAR heterotetramers in the mature brain and after plasticity^3,9,10,37,38^. It is important to note that the more flexible pattern of GluN2 subunit organization is built on the precise modular organization of the GluN1 subunit, which forms nanomodules that scale in a fashion indistinguishable from PSD-95. Thus, NMDAR organization reflects a well-organized and robust synaptic nanostructure while providing a flexible architecture (Fig. 7e).

### NMDARs and AMPARs exhibit distinct nanoscale relationships to PSD-95 nanomodules

**Fig. 8:**
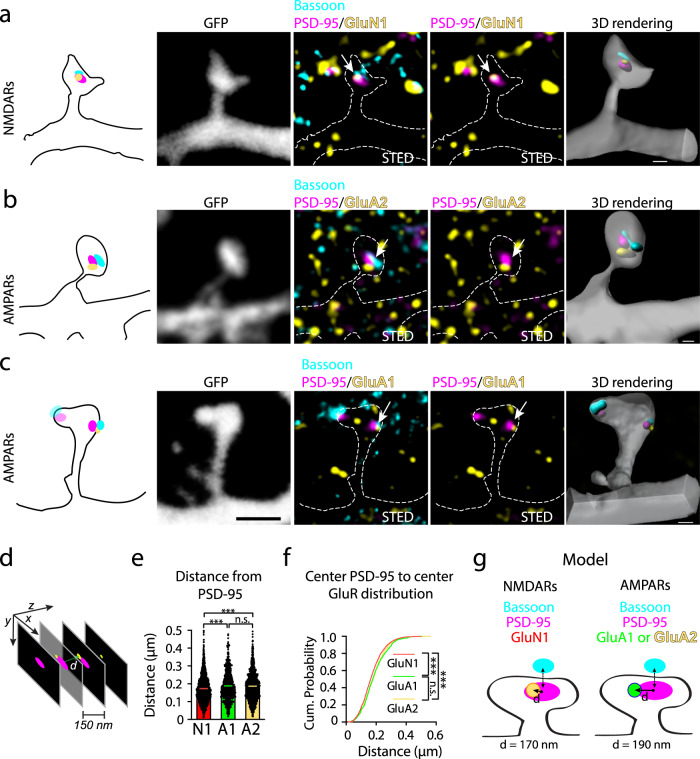
NMDARs and AMPARs are localized to distinct parts of PSD-95 nanomodules. Representative images of one (**a**, **b**) and two (**c**) nanomodule spines from DIV21 cortical neurons. Antibodies to PSD-95 (magenta, Atto-425) and Bassoon (cyan, AlexaFluor-594) were used to identify synaptic nanomodules in STED mode in GFP-labeled spines (gray, dotted line, confocal mode). Arrows indicate synaptic **a** GluN1 nanoclusters (yellow, Atto-647N), **b** GluA2 nanoclusters (yellow, Atto-647N) and **c** GluA1 nanoclusters (yellow, Atto-647N). Scale bar for projections: 1 µm (**a**–**c**). 3D rendering of indicated glutamate receptor subunit localization relative to PSD-95/Bassoon nanomodules is shown on the right. Scale bars for rendered images: 200 nm (**a**, **b**) and 300 nm (**c**). **d** Schematic of the segmentation and distance measurements (*d*, green arrow) between the centers of pre- and postsynaptic clusters that were STED resolved in *XY* (~50 nm) and *Z* (~300 nm). **e** Averages (***p < 0.0001, one-way ANOVA with Tukey’s post hoc) and **f** the cumulative probability distributions (GluN1 vs. GluA1 **p = 0.0023, GluN1 vs. GluA2 ***p < 0.0001, GluA1 vs. GluA2 *p* = 0.3623, two-tailed K–S test) of center-to-center distances between PSD-95 and co-localized GluN1 (red, *n* = 1463 clusters), GluA1 (green, *n* = 755 clusters) and GluA2 (yellow, *n* = 1457 clusters) nanoclusters. **g** Model of the sub-synaptic nanoscale localization of synaptic (PSD-95 colocalized) NMDARs and AMPARs. Measurements in **e** and **f** were performed on a per cluster basis (see “Methods”). Bars represent the mean ± SEM.

The relationship between glutamate receptors and PSD-95 at spine synapses has been the subject of intense interest^39–45^. To begin to address this relationship at the nanoscale, we measured the distances between centers of STED-resolved clusters of endogenous synaptic PSD-95 (juxtaposed to Bassoon) and colocalized nanoclusters of either NMDARs (GluN1) or AMPARs (GluA2 or GluA1; Fig. 8a–c and Supplementary Fig. 4e, f). Segmentation and subsequent quantification were performed on XYZ super-resolved nanoclusters of synaptically localized proteins within the entire imaged field (See “Methods” and Fig. 8d)^25,46^. As expected, there were no significant differences between GluA1 and GluA2 subunit-containing AMPAR localization relative to PSD-95 (Fig. 8e, f and Supplementary Fig. 4e, f). Similar results were obtained using two different combinations of secondary antibodies (PSD95- Atto-425, Bassoon- AlexaFluor-594, and GluA1- Atto 647N—Fig. 8c, and GluA1- Atto-425, PSD95- AlexaFluor-594, and Basson- Atto-647N Supplementary Fig. 10) suggesting that these findings were not due to chromatic aberration or the mix of secondary antibodies used (Supplementary Figs. 1 and 10). In contrast, the centers of PSD-95 nanomodules were significantly closer to the centers of GluN1 subunit nanoclusters than to either GluA1 or GluA2 subunit clusters both on average and over the distribution (GluN1: 173.4 ± 2.0 nm; GluA1: 189.1 ± 3.0 nm; GluA2: 186.1 ± 2.1 nm; Fig. 8e, f). Similarly, both GluN2A and GluN2B subunit nanoclusters were about the same distance from PSD-95 as GluN1 and closer to PSD-95 than GluA1 (GluN2B: 179.7 ± 2.0 nm; GluN2A: 177.0 ± 2.5 nm; Supplementary Fig. 9h, i). GluN2A subunits were closer than GluA2 subunits to PSD-95 (Supplementary Fig. 9j). These data indicate that AMPARs and NMDARs are localized to different synaptic sub-diffraction domains at individual synapses, where NMDARs are preferentially localized closer to the centers of PSD-95 and AMPARs are at the periphery of PSD-95 nanomodules (Fig. 8g; Supplementary Fig. 9a, b and h, i, j). This link between glutamate receptor modularity and spatial organization suggests that glutamate receptors are organized to optimize synaptic function^13,20,39^.

### NMDARs and AMPARs are distributed to distinct synaptic nano-domains

Among the molecules required for vesicle fusion, the presynaptic calcium sensors play a central role in regulating vesicle release kinetics^47^. The presence of the fast calcium sensor Synaptotagmin 1 (SYT1) on the membrane of synaptic vesicles is thought to underlie rapid vesicle fusion with the presynaptic membrane at the majority of central synapses^47,48^. Beyond rapid vesicle fusion, efficient synaptic transmission depends on the nanometer alignment between sites of vesicle fusion and glutamate receptors. These data suggest that the nanoscale positioning of synaptic glutamate receptors relative to glutamate release sites will impact synaptic function^1,20,21,49^. Yet, how SYT1 is organized in individual active zones and SYT1’s position relative to NMDARs and AMPARs are not known.

**Fig. 9:**
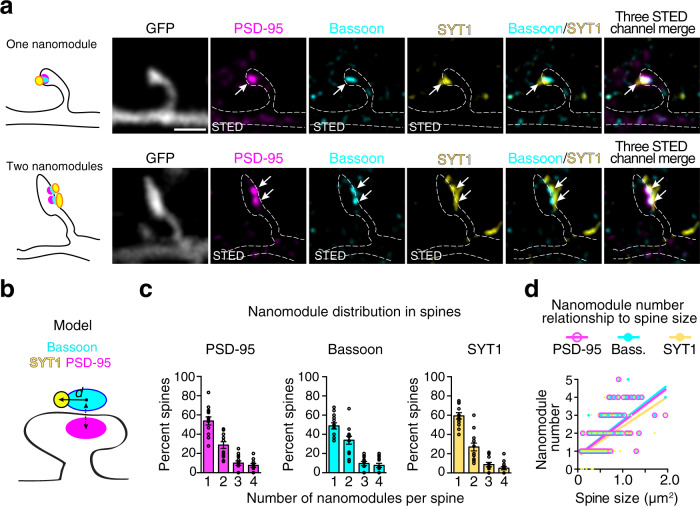
Nanoscale organization of SYT1 in dendritic spine synapses reflects the modular organization of pre- and postsynaptic adaptor molecules. **a** Representative images of one and two nanomodule spines from DIV21 cortical neurons. Antibodies to PSD-95 (magenta, Atto-425), Bassoon (cyan, Alexa-594), and SYT1 (yellow, Atto-647N) were used to identify the colocalization of the calcium sensor SYT1 with synaptic adaptor nanomodules in STED mode (arrows) in GFP-labeled spines (gray, dotted lines, confocal mode). Scale bar: 1 µm. **b** Schematic of the measurements performed to determine the organization of SYT1 relative to PSD-95-juxtaposed Bassoon nanomodules. **c** Percentage of spines containing single and multiple PSD-95, Bassoon, and SYT1 clusters (*n* = 170 spines, only spines with SYT1 were counted). **d** Quantification of the relationship between spine size (*n* = 175 spines) and the number of Bassoon (cyan line, *R*^2^ = 0.4449, slope = 1.965 ± 0.1669), PSD-95 (magenta line, *R*^2^ = 0.4463, slope = 1.892 ± 0.1602) and SYT1 (yellow line, *R*^2^ = 0.3369, slope = 1.658 ± 0.1768) nanomodules (*p* = 0.2068, Bassoon vs. SYT1; *p* = 0.3268, PSD-95 vs. Bassoon, one-way ANCOVA). Bars represent the mean ± SEM.

To begin to resolve this issue, we tested whether SYT1 might respect the modular rules of the adapter proteins and glutamate receptors by defining the relationship between presynaptic SYT1 and spine size. We stained DIV21-25 neurons with antibodies for endogenous PSD-95, Bassoon, and SYT1 (Fig. 9a). This allowed us to identify SYT1 that colocalized with Bassoon juxtaposed to PSD-95 (Fig. 9b). Consistent with the importance of SYT1 for synaptic transmission, we found that SYT1 colocalized with Bassoon at 97% of spines, and SYT1 clusters co-distributed with Bassoon and PSD-95 nanomodules at synapses (Fig. 9c). Notably, the number of SYT1 puncta scaled with spine size indistinguishable from Bassoon nanomodules (Fig. 9d). These data indicate that SYT1 follows the rules of modularity at excitatory synapses^25^.

What is SYT1’s nanoscale relationship to AMPARs and NMDARs? One possibility is that SYT1 could be localized at the active zone to match the positioning of the AMPAR in the PSDs. Such nanoscale arrangement might be necessary for efficient activation of AMPARs that have low affinity for glutamate^1,21,49^. The presence of such functionally specialized zones within a single synapse is suggested by both spatiotemporal modeling of glutamate in the cleft and the flexible nanoarchitecture of active zone and PSD proteins^20,25,36,41,42,44^. Alternatively, calcium sensors might be organized independently of the glutamate receptors with release sites stochastically distributed within the active zone.

To explore these possibilities, we measured the distance between the centers of Bassoon nanomodules and the centers of colocalized SYT1 nanomodules (Fig. 9b). In order to identify synapses, only Bassoon nanomodules that were juxtaposed to PSD-95 nanomodules were selected. At synapses, the center-to-center distances between 3D-segmented SYT1 and Bassoon nanoclusters (185 nm ± 2.0 nm) closely matched the AMPARs—PSD-95 center-to-center distances (GluA1 = 189 ± 3.0 nm, GluA2 = 186 ± 2.1 nm). Thus, similar to AMPAR localization at the PSDs, SYT1 is localized toward the edges of Bassoon marked active zones.

**Fig. 10:**
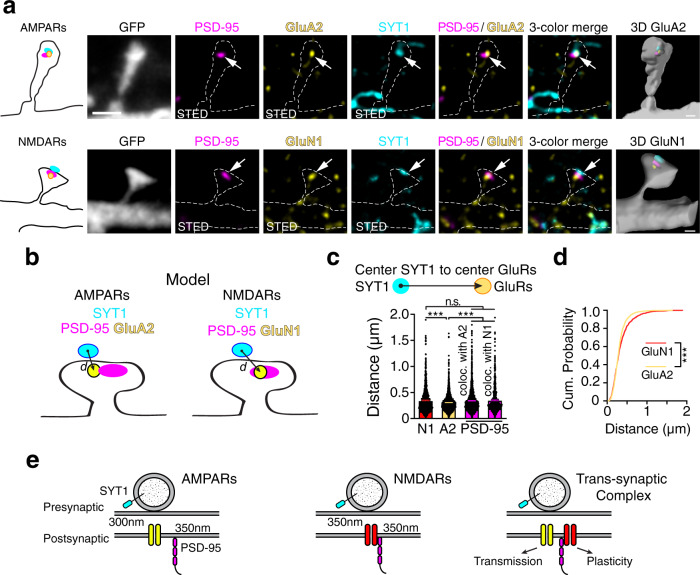
NMDARs and AMPARs are distributed to distinct trans-synaptic nano-domains. **a** Representative images of one nanomodule spines from DIV21 cortical neurons immunostained for endogenous PSD-95 (magenta, Atto-425), SYT1 (cyan, AlexaFluor-594), and either AMPARs (GluA2, yellow, Atto-647N, top) or NMDARs (GluN1, yellow, Atto-647N bottom). Arrows show the juxtaposition between the SYT1 and synaptic (colocalized with PSD-95) AMPAR and NMDAR nanomodules in STED mode in the GFP-labeled spines (gray, dotted lines, confocal mode). Scale bar: 1 µm. **b** Schematic of the measurements of the center-to-center distances between SYT1 and synaptic (PSD-95 colocalized) NMDARs (GluN1) and AMPARs (GluA2). **c** Averages (***p < 0.0001, one-way ANOVA with Tukey’s post hoc) and **d** the cumulative probability distributions (***p < 0.0001, two-tailed K–S test) of center-to-center distances between SYT1 and synaptic (PSD-95-colocalized) GluN1 (red, *n* = 921 clusters) or GluA2 (yellow, *n* = 1573 clusters). Distances between the centers of GluA2 containing PSD-95 nanomodules (*n* = 1340 clusters) or GluN1 containing PSD-95 nanomodules (*n* = 780 clusters) and SYT1 are shown on the same graph. **e** Model of how the nanoscale sub-synaptic organization of the functional pre- and postsynaptic components of a synapse might be optimized for synaptic function. Measurements in **c** and **d** were performed on a per cluster basis (see “Methods”). Bars represent the mean ± SEM.

Modeling suggests that to optimize synaptic transmission, AMPARs might be located closer to the fast SYT1 calcium sensor than the NMDARs are^21,49^. The localization of SYT1 relative to PSD-95 and the position of NMDARs and AMPARs within individual PSD-95 nanomodules was determined in GFP-transfected DIV21-25 neurons labeled with antibodies against endogenous PSD-95, SYT1, and either GluN1 or GluA2 subunits of NMDARs and AMPARs (Fig. 10a). The distances between SYT1 and synaptic NMDARs, AMPARs, or PSD-95 were determined using nearest neighbor segmentation of STED-resolved nanoclusters of GluN1/PSD-95 colocalized clusters or GluA2/PSD-95 colocalized clusters and the centers of SYT1 clusters^46^ (Fig. 10a). SYT1 clusters were significantly further from the centers of synaptic GluN1 clusters (349 ± 8.0 nm) than from synaptic GluA2 clusters (306 ± 4.2 nm, *p* < 0.0001, two-tailed K–S test). On average, SYT1 clusters were ~40 nm further from NMDARs than AMPARs (Fig. 10c). Thus, the distribution of the NMDARs shows that the bulk of NMDARs is located further from the SYT1 calcium sensor, but lie close to PSD-95 centers, while AMPARs at the PSD-95 periphery appear to be organized closer to SYT1 nanomodules (Fig. 10d). Consistent with this notion, center-to-center distances between SYT1 and PSD-95 nanomodules (either NMDAR or AMPAR colocalized) closely match the distances measured for GluN1 and SYT1 (GluA2 colocalized PSD-95: 347 ± 6.3 nm, *p* = 0.9967; GluN1 colocalized PSD-95: 355 ± 9.2 nm, *p* = 0.9475), but are significantly longer when compared to GluA2 and SYT1 measurements (Fig. 10c, *p* < 0.0001, one-way ANOVA).

The organization of SYT1 release sites in presynaptic terminals is closely linked to the localization of AMPARs in PSD-95 nanomodules (Fig. 10a–d). Three-dimensional reconstruction of GluA1, GluN1, and SYT1 clusters confirmed a closer apposition between SYT1 and AMPARs than between SYT1 and NMDARs (Supplementary Fig. 11a–c). Consistent with the above findings, the centers of SYT1 clusters were, on average, 35 nm closer to GluA1 clusters (387 ± 1.4 nm) than GluN1-containing nanoclusters (Supplementary Fig. 11d, 422 ± 2.1 nm, *p* < 0.0001, two-tailed Student’s t-test). The distribution of center-to-center distances between SYT1 and GluA1 or GluN1 were similar to our findings with PSD-95-colocalized GluA2 and GluN1 subunits, supporting the validity of this approach (Supplementary Fig. 11e, *p* < 0.0001, two-tailed K–S test). These data indicate that synaptic AMPARs are on average closer to SYT1 nanoclusters than are synaptic NMDARs. Overall, our findings demonstrate that synchronous release sites segregate to sub-diffraction zones, mirroring the AMPARs’ organization in spines. These data indicate the presence of specialized trans-synaptic nanomodules likely designed for a particular synaptic function (Fig. 10e).

## Discussion

It has long been proposed that nanoscale precision in synaptic organization allows for the maximal efficiency of synaptic transmission and plasticity^20^. Here we define the subunit-specific rules that guide the organization of AMPAR and NMDAR heterotetramers and relate the organization of these proteins to both spine size and presynaptic calcium sensors. Both AMPAR and NMDAR heterotetramers form modules that scale in number as spine size increases. The location of these modules is guided by function, with AMPARs closer to the fast calcium sensor SYT1 and NMDARs closer to nanomodules of PSD-95. These data support a model where AMPARs and NMDARs are positioned to maximize functional efficiency—with AMPARs located for optimal activation by glutamate and NMDARs located close to the scaffolding nexus to maximize their role in the induction of plasticity. Thus, despite their small size, single synapses likely contain specialized nanoscale sub-domains that allow for the optimization of synaptic function.

The organization of glutamate receptor heterotetramers is consistent with a nanoscale architecture that reflects the specific functions of glutamate receptor subunits and allows for flexibility likely needed for events such as synaptic plasticity. Both AMPA and NMDA receptors form nanomodules that scale with spine size, reminiscent of the modular organization of the MAGUK PSD-95^25,33^. Increasing the number of glutamate receptor clusters in a modular fashion as spines increase in size would help maintain stable NMDAR-AMPAR ratios at individual synapses in response to ongoing plasticity^50^. These data are consistent with both the changes in AMPAR and NMDAR heterotetramer number that occur following structural plasticity^5,17,30^ and the stable AMPAR/NMDAR ratio seen after synaptic plasticity^50–54^. Within this modular framework there is flexibility generated by the specific subunit composition of AMPA and NMDA receptors.

AMPAR heterotetramers appear to be organized at the nanoscale for optimal subunit function, which generates a characteristic pattern of nanoscale organization of AMPAR subunits in relation to spine size. The pattern of AMPAR organization is reflected by differences in the organization of GluA1-containing AMPARs vs. those heterotetramers that lack GluA1. Nanoclusters of GluA2 subunit-containing AMPARs overall scaled in number as spine size increased and GluA2 immunostaining was localized to the majority (89%) of spines. In contrast, GluA1-containing AMPARs (~90% of which contain GluA2 subunits and are likely GluA1/A2 heterotetramers, (Supplementary Fig. 4d)^2,26,27^ are primarily localized to large spines that contain two or more juxtaposed PSD-95/Bassoon nanomodules. These data are consistent with the model that AMPARs containing GluA1 subunits are found in larger spines and suggest that subunit composition is likely important for guiding their nanoscale localization at synapses^2^.

The GluN2B subunit of the NMDAR is linked to the induction of synaptic plasticity and maturation or development of spine synapses^3^. Consistent with this model, GluN2B subunit-containing nanoclusters were enriched in smaller spines. This organization likely establishes a distinct class of NMDAR nanoclusters, as only GluN2B-containing nanoclusters did not exhibit positive scaling with spine size. Indeed, there is a negative relationship between the GluN2B nanocluster numbers and spine size. Given the role of these NMDAR subunits in tuning synaptic function and plasticity, the small, GluN2B-only containing spines are consistent with observations that spines with GluN2B-containing NMDAR heterotetramers might represent a group of spines that are primed for synaptic plasticity^7,37,55^.

The rules governing NMDAR subunit distribution to larger spines appear straightforward and designed to allow for the flexible function of spine synapses. As spine size increases, GluN2A but not GluN2B-containing nanoclusters increase in number. These increases parallel increases in PSD-95 nanomodules and result in two types of larger spines: spines that contain GluN2A subunits or spines that contain a mixture of GluN2A and GluN2B subunit nanoclusters. These data are consistent with biochemical analyses showing a correlation between events that result in structural plasticity (LTP induction and sensory experience) and increases in levels of GluN2A-containing NMDAR density in the PSDs^30,56,57^. Defining the mechanisms that regulate the nanoscale organization of glutamate receptor heterotetramers in spines will be key for unraveling their functions at synapses.

Non-synaptic nanoclusters of AMPARs and NMDARs are abundant in spines but do not conform to the modular rules that drive the organization of synaptic nanoclusters of glutamate receptor heterotetramers. GluA2 subunit non-synaptic nanoclusters were found in most spines, while non-synaptic GluA1 and GluN1 subunit nanoclusters were found in only about 50% of spines. The abundance of these non-synaptic glutamate receptor nanoclusters is consistent with the presence of extrasynaptic, laterally diffusing glutamate receptors that may serve as a reservoir for delivery to and exchange with synaptic nanoclusters, particularly glutamate receptors containing GluA2 subunits^18,19,58^. This notion is further supported by our cLTP experiments in which we observed that NMDAR-dependent spine enlargement is linked to the formation of new GluA2 nanoclusters at synaptic sites, similar to what was previously reported for PSD-95^25^. However, trafficking and nanorganization of GluA1 vs. GluA2 containing AMPARs to spines after plasticity is likely more complex especially given the findings that GluA1 and GluA2 subunits’ synaptic localization is differentially controlled by their N-terminal domains^59^. Non-synaptic nanoclusters also likely represent the pool of internalized and recycling glutamate receptors^17^. Consistent with these functions, non-synaptic nanoclusters of glutamate receptor heterotetramers were smaller than synaptic clusters^13^. The architecture of the spine, with clusters of non-synaptic and synaptic receptors that follow different sets of rules, highlights the importance of examining the relationship between neuronal morphology and the localization of pre- and postsynaptic proteins to understand the nanoscale organization of the synapse.

Exploration of the nanoscale organization of the synapse has revealed a complex and ordered network of pre- and post-synaptic proteins. Post-synaptic glutamate receptors and MAGUKs, and pre-synaptic Bassoon, vGlut1, SYT1, and Synaptophysin appear to share a modular organization where the number but not size of puncta of these proteins increase with increases in spine size^25,41,42,44^. Importantly most spines (60%) have only one nanomodule, with few spines having more than two of these paired pre- and postsynaptic nanoclusters. Ultrastructural EM analysis of spines demonstrates the presence of spines with perforated postsynaptic densities and corresponding clusters of presynaptic features^60–62^. Moreover, changes in the number of perforated spines have been observed following the induction of structural plasticity in vivo^63^. Photostimulation induced structural plasticity at individual spines results in rapid increases in the number of perforated and segmented spines with a time course similar to changes in the nanoscale organization of synaptic proteins^25,64^. It will be of significant interest to determine the relationship between the nanoscale composition of synaptic proteins and the synaptic ultrastructure which will likely require the development of new methods including fixation protocols^65^.

While both NMDARs and AMPARs reflect the modular organization of PSD-95 in spines, they have distinct nano-localizations within individual PSD-95 nanomodules. NMDAR nanoclusters are preferentially localized toward centers of PSD-95 nanomodules, whereas AMPAR nanoclusters are found at the periphery of PSD-95 nanomodules. These results support the model that NMDARs, but not AMPARs, are found at the core of PSDs^13,39^. This pattern of arrangement is also consistent with the differential stability of NMDARs and AMPARs at synapses. Lateral diffusion of AMPARs at synapses controls the number of AMPARs at PSDs and in plasticity^19,58^; therefore, their peripheral localization might enable the rapid exchange of AMPARs into and out of synapses. On the other hand, NMDARs appear to be much more stable at synapses, consistent with their interior nanoscale localization^15^. These data suggest that the nanoscale organization of the synapse is highly ordered.

Synaptic function is likely tightly linked to the nanoscale organization of AMPA and NMDA receptor sub-types^13,20,41,42,66^. Our data suggest that these proteins are highly ordered and localized in predictable ways. However, how such nanoscale patterning occurs remains unclear. Given that different subunits follow specific rules, it is likely interactions mediated directly by the receptor subunits themselves play key roles in the nanoscale patterning of these proteins. Attractive candidate mechanisms for establishing and regulating nanoscale synaptic organization include intracellular interactions^3,17,45^, extracellular interactions^59,66–69^, and liquid–liquid phase separation^70–72^. How these mechanisms might establish functionally distinct synaptic sub-domains is currently an open question and that will require further investigation.

What is the importance of the distinct AMPAR and NMDAR nano-organization at synapses? Segregating AMPARs and NMDARs might allow for more flexibility in synaptic function. For example, the nanoscale localization of AMPARs at the periphery of individual PSD-95 nanomodules is tightly matched to the nanoscale organization of the fast SYT1 calcium sensor, essential for synchronous vesicle fusion, to the edges of Bassoon nanomodules. In contrast, NMDARs and SYT1 nanoclusters are organized somewhat independently of each other. This tight structural arrangement between synchronous sites of vesicle fusion and AMPARs is likely necessary for efficient AMPAR activation following the release of glutamate—due to their low affinity for glutamate^1,21,49^. The importance of such a precise nanoscale alignment between the sites of fusion and AMPARs was suggested by experiments in which the light-driven recruitment of AMPARs to synapses was insufficient to increase synaptic strength^73^. Consistent with our structural findings, the activation of AMPARs is much better coupled to the release of a quantum of glutamate than NMDARs^13^. The weaker coupling of NMDAR heterotetramers could be explained by a poorer alignment between NMDARs and SYT1. Alternatively, perhaps the differential localization of AMPAR and NMDAR heterotetramers reflects specialized nanoscale signaling hubs that contain relevant molecules required for specific synaptic functions and plasticity. Regardless, these data illuminate the robust rules of modularity that generate an exquisitely detailed organization of pre- and postsynaptic structures endowing synapses with the multifunctional but flexible nanoarchitecture necessary for neurotransmission and synaptic plasticity.

## Methods

### Animals

All animal studies were approved by the Institutional Animal Care and Use Committee guidelines at Thomas Jefferson University in accordance with the US National Institutes of Health guidelines. Long-Evans E17-18 male and female rat embryos from timed pregnant animals purchased from Charles River Laboratories Inc. (Wilmington, MA) were used to make primary cortical neuron cultures (see below).

### Primary cortical neuron culture preparation

Dissociated cortical neurons were prepared from embryonic day 17-18 (E17-18) rat cerebral cortex as described previously^25,74–76^ and cultured in Neurobasal medium (cat#: 21103049, Thermo Fisher Scientific) supplemented with 1x B27 (cat#: A3582801, Thermo Fisher Scientific), 2 mM glutamine (cat#: 25030081, Thermo Fisher Scientific) and penicillin-streptomycin (cat#: 15140122, Thermo Fisher Scientific). Neurons were plated on poly-D-lysine (cat#: 354210, Corning, Corning, NY) and laminin (cat#: 354232, Corning, Corning, NY) coated glass coverslips (12 mm, #1.5; Cat#: 64-0712, Warner Instruments, Camden, CT). Neurons were plated at 150,000/well in 24-well plates and were maintained in a humidified 37 °C incubator with 5% CO2 until DIV21-25.

### Neuronal transfection

Neurons were transfected at day in vitro 10 (DIV10) as previously described^25,68,69,74,75^ using Lipofectamine 2000 (cat#: 11668027, Thermo Fisher Scientific). EGFP under control of human ubiquitin promoter (pFUg-EGFP) was used as a cell filling dye to visualize neuronal morphology^25,74^. Briefly, the conditioned medium was first collected from plated neurons and replaced with 300 µl of Neurobasal medium without any supplements (per one well of 24-well plate). 100 µl of transfection mix containing 0.5 µl of Lipofectamine 2000 and 200 ng of pFUg-EGFP plasmid was then added per well of a 24-well plate. Neurons were incubated with the transfection cocktail at 37 °C for 2 h. After 2 h, the transfection medium was replaced with 500 µl of warmed conditioned medium, and neurons were placed in a humidified 37 °C incubator until DIV21-25, at which point they were used for immunocytochemistry and STED.

### Immunocytochemistry

For immunocytochemistry, cultured cortical neurons were fixed between DIV21 and DIV25 in 4% paraformaldehyde (PFA)/2% sucrose supplemented with 0.0025% glutaraldehyde (cat#: 16000, Electron Microscopy Sciences, Hatfield, PA) in PBS for 8 min at room temperature. Fixed neurons were then washed once in PBS, followed by a 15 min incubation at 4 °C in 1 mg/ml sodium borohydride solution (cat#: 213462-25G, Millipore Sigma, St. Louis, MO) diluted in PBS. Coverslips were then washed three times in PBS, blocked, and permeabilized for 2 h at room temperature in 1% ovalbumin (cat#: A5503, Millipore Sigma) and 0.2% gelatin from cold-water fish (cat#: G7041-100G, Millipore Sigma) in PBS containing 0.01% saponin (cat#: 47036, Millipore Sigma). Neurons were then stained for 2 h at room temperature or overnight at 4 °C with the indicated primary antibodies, washed three times in PBS, and then immunostained with corresponding secondary antibodies for 45 min at room temperature. After washing three times in PBS, coverslips were mounted with MOWIOL mounting medium and used for confocal and STED imaging 24 to 48 h post-mounting.

### Antibodies

All primary and secondary antibodies were profiled in our previous publications and were reported to be specific^25,68,69,74–77^.

Primary antibodies used were: mouse monoclonal (IgG2A) anti-PSD-95 clone K28/43 (1:200, Neuromab, UC Davis, Davis, CA), mouse monoclonal IgG1 anti-PSD-95 clone 7E3-1B8 (1:250, Thermo Fisher Scientific, Waltham, MA, cat#: MA1-046) anti-guinea pig polyclonal anti-Bassoon (1:300, Synaptic Systems, Gottingen, Germany, cat# 141 004), rabbit polyclonal anti-Bassoon (1:300, Synaptic Systems, cat #: 141 003), chicken anti-GFP (1:2000, Abcam, Cambridge, MA, cat# ab13970), rabbit monoclonal anti-GluN1 (1:500, AB9864, Millipore Sigma), mouse monoclonal (IgG2A) anti-GluN2A clone N327/95 (1:250, Neuromab), mouse monoclonal (IgG2B) anti-GluN2B clone N59/36 (1:250, Neuromab), mouse monoclonal (IgG1) anti-GluA1 clone N355/11 (1:250, Neuromab), rabbit polyclonal anti-GluA2(1:500, Synaptic Systems, cat#: 182 103), mouse monoclonal (IgG2A) anti-Synaptotagmin 1 (1:500, Synaptic Systems, cat#: 105 011).

Secondary antibodies used were: Goat anti mouse IgG2A Atto 425 (1:250, Rockland, Inc., cat# 610-151-041), Goat anti-mouse IgG1 Atto-425 (1:250, Rockland, cat#: 610-151-040), Goat anti-rabbit Atto 425 (1:250, Rockland, cat#: 611-151-122), Goat anti-mouse IgG1 Atto-647N (1:500, Rockland, Inc., cat # 610-156-040), Goat anti-mouse IgG2A Atto-647N (1:500, Rockland, cat#: 610-156-041), Goat anti mouse IgG2B Atto-647N (1:500, Rockland, cat#: 610-156-042), Goat anti-rabbit Atto-647N (1:500, Rockland, Inc., cat # 611-156-122), Goat anti-mouse IgG1 AlexaFluor-594 (1:500, Jackson ImmunoResearch, cat# 115-587-185), Goat anti-mouse IgG2A AlexaFluor-594 (1:500, Jackson ImmunoResearch, cat# 115-585-206), Donkey anti guinea pig AlexaFluor-594 (1:500, Jackson ImmunoResearch, cat # 706-586-148). Donkey anti-Rabbit AlexaFluor-594 (1:500, Jackson ImmunoResearch cat # 711-585-152).

### Chemical LTP

NMDAR-dependent cLTP was induced by treatment of DIV 21-25, GFP-transfected neurons with glycine (200 µM) as described^25,28,29^. Neurons were placed in artificial cerebrospinal fluid (ACSF, 143 mM NaCl, 5 mM KCl, 2 mM CaCl_2_, 1 mM MgCl_2_, 30 mM glucose and 10 mM HEPES, pH 7.4) containing 0.5 µM TTX, 1 µM strychnine and 20 µM bicuculline. After imaging baseline morphology for 15–30 min at 6 min intervals, neurons were treated with 10 mL of glycine-stimulating solution (143 mM NaCl, 5 mM KCl, 2 mM CaCl_2_, 0 mM MgCl_2_, 30 mM glucose, 10 mM HEPES, pH 7.4, 0.5 µM TTX, 1 µM strychnine, 20 µM bicuculline and 200 µM glycine) for 3–5 min, followed by 10 mL of 0 mM MgCl_2_ containing ACSF (143 mM NaCl, 5 mM KCl, 2 mM CaCl_2_, 0 mM MgCl_2_, 30 mM glucose, 10 mM HEPES, pH 7.4, 0.5 µM TTX, 1 µM strychnine, and 20 µM bicuculline). To block cLTP and spine enlargement, 50 µM D-APV (D-2-amino-5-phosphonovalerate) and 10 µM of MK-801 were included in the solutions described above. Control neurons were imaged in ACSF and not subjected to glycine. Neurons were imaged for 3 h at 6 min intervals to monitor long-term changes in spine size. Spines were classified as potentiated only if their area increased by ≥10% over baseline immediately following glycine treatment and remained enlarged throughout the entire imaging period.

### Imaging–STED nanoscopy

Three-color STED imaging of endogenous synaptic proteins was performed as described previously^25^. Briefly, a Leica TCS SP8 gated STED (gSTED) 3X super-resolution system (Leica Microsystems) equipped with a tunable white light laser, CW 592 and 660 nm depletion lines, and a pulsed 775 nm depletion line was used for image acquisition. Resonance scanning (8000 Hz), gated HyD detectors (set at 100–200%), and 100× oil immersion objective (Leica) with 5–10× zoom to obtain desired pixel size (25 nm) was used to acquire stacks at 150 nm image intervals. All data shown were imaged using 3X STED. Proteins labeled with Atto-647N or Alexa-594 secondary antibody conjugates were acquired using gSTED with HyD detectors adjusted between 0.2/0.3 to 6 ns. First, Atto-647N labeled endogenous proteins were excited with the 647 nm laser (5–15% maximal laser power), and second, Alexa-594 labeled endogenous proteins were excited using the 594 nm laser (5–12% maximal power). The pulsed 775 nm depletion line (set at 10–15% of maximal laser power for Atto-647N fluorophore and 35–50% of maximal laser power for Alexa-594 fluorophore) was used to generate STED with a resolution of ~50 nm, measured by determining the FWHM of puncta^25^ (Supplementary Fig. 2, see below). Lastly, proteins labeled with Atto-425 fluorophore were visualized by exciting with 442 nm line (12–15% power), and the CW 592 nm line (50–65% power) was used to generate STED. For this line, non-gated HyD detectors set to 250% gain were used to obtain super-resolved images (~80 nm *XY* resolution, measured by the FWHM of puncta). For *Z* depletion, 15% of the 775 and 592 nm depletion line power was re-directed to the *Z* donut to achieve an image *Z*-resolved at ~250–300 nm^25^.

For resolution determination, single Alexa 594 fluorophores, identified as the smallest single puncta in the background, were imaged in *XY* in confocal and STED with 30% *XY* 775 nm depletion either with 0% *Z*-Depletion power or 15% *Z*-Depletion power (Supplementary Fig. 2a). Full width at half maximum (FWHM) was measured from Gaussian fits of individual line plot profiles from lines drawn horizontally through puncta centers (white arrows, Supplementary Fig. 2a; individual line plot profiles: gray lines Supplementary Fig. 2b; average Gaussian fit: green lines Supplementary Fig. 2b). The point-to-point resolution was determined using GATTA 50 nm spaced Alexa 647 beads (GATTAQUANT GMBH, Grafelfing, Germany) imaged in confocal and STED with 775 nm depletion with 0% *Z*-depletion. Of note, the Alexa 647 dye is different than the dye used in immunolabeling (Atto 647N) and thus the resolution may vary slightly. The two GATTA beads spaced 50 nm apart were measured in STED with or without deconvolution (Supplementary Fig. 2d). Peak-to-peak distances were calculated from line plot profiles from lines drawn through the centers of both resolved beads (Supplementary Fig. 2d, e, raw data - gray traces, average - red line). To determine the dual-color STED point-to-point resolution, GATTA Dual color 70 nm Alexa 647/ Alexa-594/ Alexa 647 spaced beads were imaged in confocal and STED with 775 nm depletion laser with 0% *Z*-depletion (Supplementary Figure 2g, h). Peak-to-peak distances were calculated from line plot profiles of the three beads by passing a line through all three peaks of the dual color GATTA beads (Supplementary Fig. 2g, h).

Time-lapse imaging of live neurons was performed with either the confocal spinning disk or Leica SP8. The confocal spinning disk was equipped with a Yokogawa CSU-10 and a Hamamatsu EM-CCD digital camera attached to an inverted Lecia microscope and controlled by Metamorph software 7.10. 2–4 μm image stacks were collected with an optical sectioning of 0.2 µm using 100× (spinning disk), or 0.35 µm using 63× (Leica SP8) oil immersion objectives. Adaptive focus control was utilized throughout the duration of time-lapse imaging. Following imaging, neurons were immediately fixed, stained, and imaged using three-color STED as previously reported^25^.

### Image processing and deconvolution

Detailed methodology for image processing, along with the FWHM measurements, is described in the second supplemental figure in Hruska et al.^25^. Briefly, images collected using the SP8 Leica gSTED from cultured neurons were deconvolved as stacks using Huygens deconvolution software (SVI, Hilversum, Netherlands) by specifying the point spread function (PSF, Leica SP8/DM6000/100× objective, imaging wavelength), optical sectioning, *X*, *Y*, and *Z* pixel resolution^78^. Deconvolution was performed separately for each channel using a maximum of 40 iterations. Each image was deconvolved using the same parameters. The effect of deconvolution on nanocluster sizes is minimal since the clusters sizes’ are larger than the reported resolution.

### Image analysis

Image analysis was conducted off-line using Fiji Image J (https://imagej.net/Fiji) and built-in macros, as described below.

For nanomodule identification, super-resolution analysis of synaptic cluster localization in dendritic spines was performed on a per spine basis. Images of spines, acquired at confocal resolution (~250–300 nm), were detected visually, and Gaussian blur (2-pixel value) was applied to filter out noise. Individual spines were converted to binary masks by thresholding the resulting EGFP image. Spine edges were determined by thresholding EGFP to the mean +2 × SD (Standard Deviation) of the 1024 × 1024 pixel area corresponding to the entire image field. Nanoclusters of synaptic proteins (acquired in STED super-resolution) were identified by binarizing each channel separately using intensity thresholds. Thresholds were defined as the mean +2 × SD of intensity values of a 50 × 50 image pixel area. For three-color STED using gated detectors, clusters were defined as a minimum of 10 and a maximum of 100 continuous pixels corresponding to an area of 0.002–0.15 µm^2^. The separation between neighboring STED resolved clusters were identified from the line intensity profiles of nearby clusters and was defined as the mean +1.5 × SD of a local 50 × 50 pixel area that approximately corresponded to the maximum size of a spine head. The resulting thresholded nanomodules were used to determine whether these modules colocalized with individual spines. Only spines with clearly identifiable PSD-95 and/or Bassoon clusters were included in the analysis.

For 3D STED cluster assignments, outlines of spines were determined in individual Z sections of thresholded images. ROIs (region of interest) of each thresholded spine head were used to manually assign STED-resolved puncta to spines. PSD-95, AMPARs, and NMDARs clusters were assigned to a spine if the thresholded pixel areas were entirely within the spine head ROI. Bassoon and SYT1 clusters were assigned to a spine if the thresholded pixel areas either entirely or partially overlapped with the spine head ROI. Spine ROI colocalization of each cluster was made independently for each *Z* section. Orthogonal views of the overlaid image stacks were used to verify that individual clusters colocalized with the spine ROI in the *Z* plane. Finally, image stacks were overlaid and filtered by an edge-preserving algorithm in Imaris software (Bitplane AG 8.3.1). High-contrast images of puncta within the area that corresponded to the size of the spine head and shaft (approximately 100 × 100 pixels) were projected in Imaris to generate high-contrast volume rendered images. Volume rendering was performed for each channel separately using a two-voxel separation between thresholded objects. Thresholded clusters that did not colocalize with the area of the spine were discarded. Data for spine analysis of module number represent observations and were acquired and analyzed with an experimenter blinded to the condition during analysis.

Colocalization and nearest neighbor analysis of 3D STED-resolved synaptic clusters were performed on a per-cluster basis using the entire area of an image (1024 × 1024 pixel format). Segmentation and subsequent measurements of distances of segmented clusters were performed using the DiAna plugin in Image J that enabled the analysis to be done in an automated way^46^. Segmentation of all synaptic clusters was performed using the local maxima method combined with user-defined thresholds^46^. Local maxima in 3D were identified using a radius of 3 pixels in the *XY* plane and 2 pixels in the *Z* plane. Since deconvolved images were used for this analysis, the noise was set to zero. Local thresholds were determined by thresholding individual 16-bit images (1024 × 1024 pixel format) in each channel and were determined to be between 10,000 and 20,000 arbitrary units (AU). The maximum radius of segmented clusters was set to 8 pixels (individual pixel sizes in our images were set to 23–25 nm to allow maximum resolution of ~50 nm). The standard deviation for Gaussian fit and threshold calculation was set to 1.5. Minimum and maximum voxel sizes were set to 3 and 20,000, respectively. Distance analysis of segmented clusters is based on classical Euclidean distance computation^46^. For trans-synaptic cluster measurements, we implemented center-to-center distances where, for each object from one image (channel 1), the center-to-center distances with all objects from another image (channel 2) were computed in 3D, and closest neighbor distances were reported. For these measurements, we only included objects that had clear juxtaposition between pre- and post-synaptic markers. For post-synaptic only measurements (PSD-95 with either AMPARs or NMDARs) or pre-synaptic only measurements (Bassoon with SYT1), we calculated distances only for colocalized objects in order to determine the positioning of synaptic AMPARs or NMDARs, and SYT1. For GluN2A-GluN2B distance measurements (Supplementary Fig. 9e–g), we calculated distances only between GluN2A and GluN2B clusters that were both colocalized with a PSD-95 nanomodule within spine heads.

For chromatic aberration analysis, neurons transfected with GFP were imaged at DIV 21–25 and stained for endogenous Bassoon with three different antibodies against the same primary antibody (Rb Atto-425, Rb AlexaFluor-594, Rb Atto-647N) (Supplementary Fig. 1a). Triple-colocalized puncta were selected for analysis based on whether they had each of the three colors at similar brightness. The peak-to-peak distances between each secondary antibody was determined from line plot profiles of each of three secondaries, measured by averaging the line plot profiles of four lines passing through the center of the triple-colocalized puncta for each of the three secondaries (Supplementary Fig. 1a, indicated by the white dashed line). In the axial plane, peak-to-peak distances were determined from the line plot profiles measured by passing a line through the center of each triple-labeled Bassoon puncta along the *Z*-plane (Supplementary Fig. 1d, dashed black line).

### Statistics and reproducibility

Data were acquired and analyzed based on the standards in the field; however, no method of randomization was used to determine how samples were allocated to experimental groups and processed. Unless otherwise stated, data in figures and text are expressed as means ± SEM. All data points collected were included for analysis. Statistics were performed using GraphPad Prism 8.0. Statistical significance of the differences among groups was determined by one-way analysis of variance followed by post hoc tests as described in individual figure legends, or by two-tailed Student’s t-test when testing differences between two conditions. Kolmogorov–Smirnov (K–S) tests were used to test differences between non-parametric probability distributions. *P* values less than 0.05 were considered statistically significant. For *p* values less than 0.0001 or greater than 0.9999, we are providing a range and not the exact number. The data distribution was assumed to be normal, but this was not formally tested. Sample sizes were determined based on our previous publication^25,68,69,74–77^. We also performed power analysis using the power of 0.8–0.95 for medium and large effect sizes (as found in our preliminary studies) in G power software 3.1 to validate our sample sizes for statistical analyses. Group differences in variance were tested for each data set and determined to be similar. Unless stated otherwise, statistical tests were conducted on a per spine basis. Statistical tests for center-to-center distances between 3D STED clusters were performed on a per cluster basis. Data were collected from a minimum of nine different neurons acquired from three independent transfection experiments unless otherwise stated in figure legends.

### Reporting summary

Further information on research design is available in the Nature Research Reporting Summary linked to this article.

## Supplementary information

Supplementary Information

Reporting Summary

## Data Availability

Data supporting the findings of this study are available within the article, its Supplementary Information and Source Data files. Additional information and relevant data will be available from the corresponding author upon reasonable request. The Source Data underlying Figs. 1b–i, 2e–i, 3b–h, 4b–f, 5b–h, 6b–g, 7b–d, 8e, f, 9c, d, 10c, d and Supplementary Figs. 1b, c, e, f, 2b, c, e, f, h–k, 4b–f, 6a, b, 9c–j, 10c, d, 11d, e are provided with this paper. Source data are provided with this paper.

## Source data

Source Data
