# Supplementary material for: Nanoscale rules governing the organization of glutamate receptors in spine synapses are subunit specific
Source: Nat Commun. 2022 Feb 17;13:920. doi: 10.1038/s41467-022-28504-4 (PMC8854560; doi:10.1038/s41467-022-28504-4)
Supplement: Supplementary file 1 — Supplementary Information [file 41467_2022_28504_MOESM1_ESM.pdf]

Supplementary Information:

Title: Nanoscale rules governing the organization of glutamate receptors in spine synapses are subunit specific.

Authors: Martin Hruska[1,#], Rachel E. Cain[2,#], Matthew B. Dalva[2*]

#: Authors contributed equally to this work

Addresses:

1: Department of Neuroscience, Rockefeller Neuroscience Institute, West Virginia University, 108 Biomedical Road, Morgantown, WV 26506

2: Department of Neuroscience and Jefferson Synaptic Biology Center, Sidney Kimmel Medical College at Thomas Jefferson University, 233 South 10th Street, Bluemle Life Sciences Building, Room 324, Philadelphia, PA 19107 USA

Contact: Matthew B. Dalva*

Email: matthew.dalva@jefferson.edu

Tel: 215-503-0997

Supplementary Figure 1:

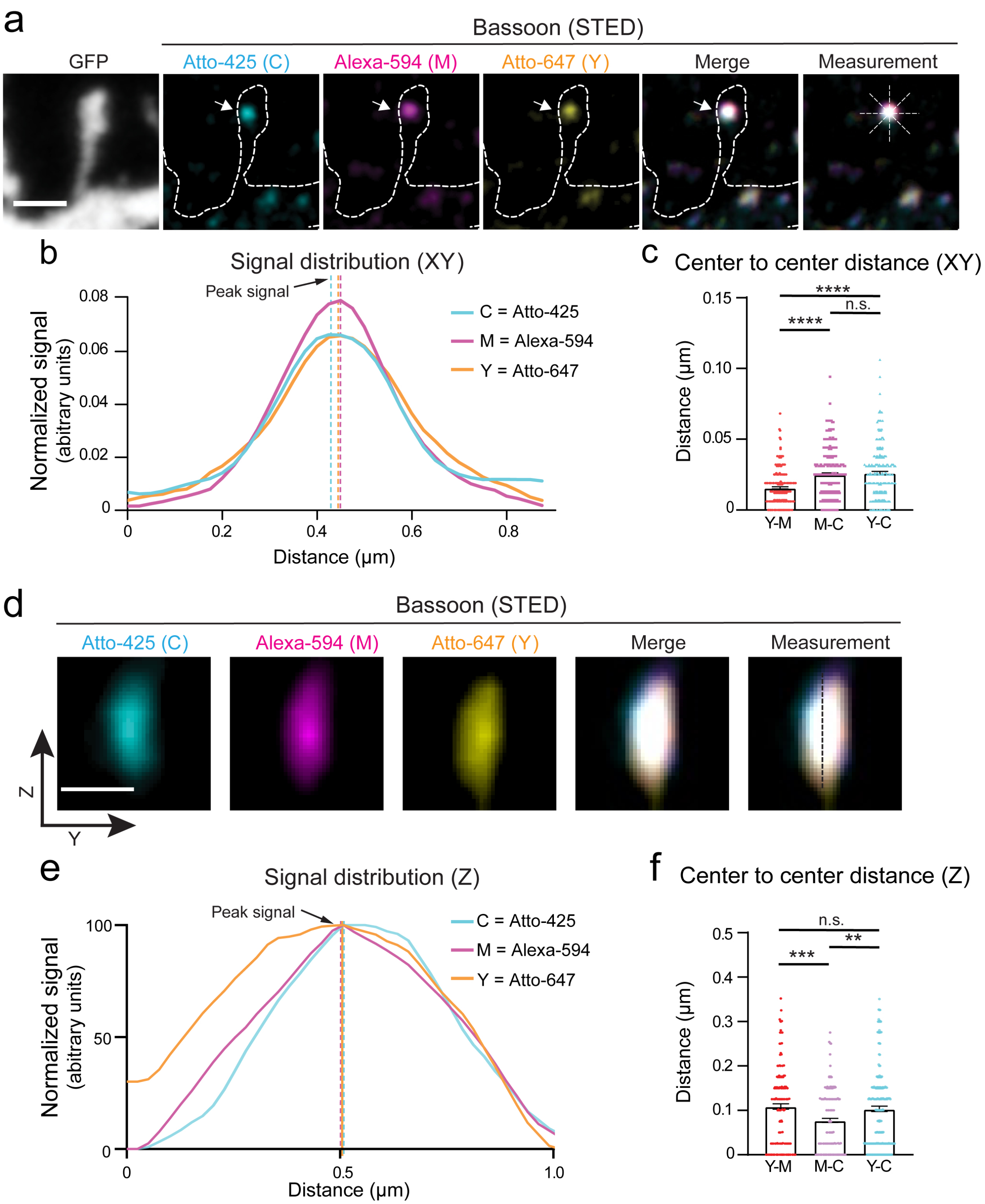

Supplementary Figure 1. Chromatic aberration is below the resolution limit. a, Representative image of a dendritic spine from a DIV21 cortical neuron transfected with GFP and stained for three different secondary antibodies against one primary antibody targeting endogenous Bassoon (Atto-425: cyan, AlexaFluor-594: magenta, Atto-647N: yellow). The average line plot profile for each secondary antibody was measured on individual triple-colocalized puncta by drawing lines in four different directions through the center of a triple-colocalized puncta to account for orientation differences (four white dashed lines). **b,** Corresponding line profiles from each colocalized cluster were then averaged and the quantification was performed on a per puncta basis. Example of four-line average profiles for each secondary antibody of the triple-colocalized Bassoon puncta shown in **a**. Center-to-center distances were measured based on the average peak signal from the four line measurements. **c,** Average (****p<0.0001, one-way ANOVA with Tukey's post hoc) XY center-to-center distances within triple-colocalized puncta (n=250 triple-colocalized puncta) between Yellow and Magenta (Y-M) (15.6 $\pm$ 0.8nm), Magenta and Cyan (M-C) (25.1 $\pm$ 1.1nm) and Yellow and Cyan (Y-C) (26.0 $\pm$ 1.3nm) are below the XY resolution (~50nm). **d,** Representative image of a triple-colocalized cluster in YZ. Line plot profiles in the Z plane were extracted by passing a line through the center of each triple-colocalized puncta in the Z dimension (black dashed line). **e,** Example line plot profiles of each secondary antibody of a triple-colocalized Bassoon puncta shown in **d**. Center-to-center distances were measured in Z based on the localization of the peak signal in the Z plane. **f,** Average (p=0.1153, one-way ANOVA with Tukey's post hoc) peak-to-peak distances in Z within triple-colocalized puncta (n=217 triple-colocalized puncta) between Yellow and Magenta (Y-M) (108.8 $\pm$

5.9nm), Magenta and Cyan (M-C) (77.3 ± 4.7nm) and Yellow and Cyan (Y-C) (103.2 ± 6.0nm) are below the Z resolution limit (~300nm). Bars represent the mean ± SEM. Scale bar in **(a)**: 1μm **(d)**: 0.5um.

Supplementary Figure 2:

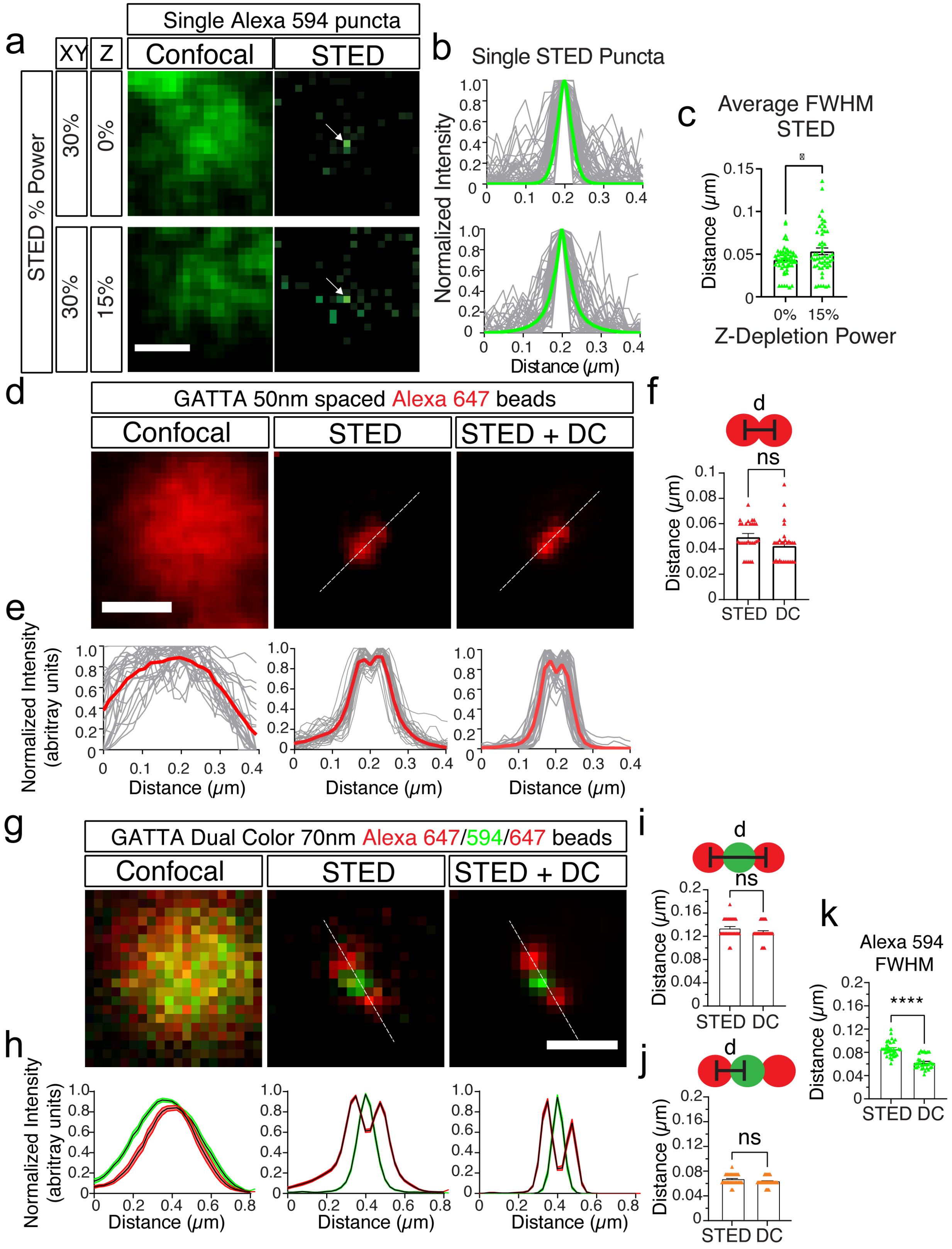

Supplementary Figure 2. Maximum and point-to-point resolution with STED. a, Representative confocal and STED images of single fluorophores (arrows) of AlexaFluor-594 imaged in XY. The 775nm pulsed laser AOBS was set to 30% with either 0% 775nm laser redirection to Z-doughnut for the maximum XY STED (top panels) or 15% 775nm laser redirection to Z- doughnut (lower panels) **b,** Line plot profiles of single fluorophores (light grey) with average Gaussian fit (green) for fluorophores imaged with 0% (top panel, n=56 puncta) and 15% (bottom panel, n=51 puncta) Z-Depletion power. **c,** Average FWHM for fluorophores imaged with 0% vs. 15% Z-Depletion power (43 ± 2.1nm vs. 53 ± 3.9nm, *p=0.0268, two-tailed Student's t-test). **d,** Representative image of GATTA 50±5nm spaced AlexaFluor-647-labeled bead imaged in confocal, STED with Gaussian blur (0.8 pixel), and STED + deconvolution (DC). **e,** Raw (grey) and average (red) line plot profiles of 50nm spaced AlexaFluor-647 beads in confocal (n=27), STED (n=35), and STED + DC (n=35). Pixel size: 15nm. **f,** Average peak-to-peak distance (p=0.063, two-tailed Student's t-test) calculated from line plot profiles of 50±5nm spaced GATTA beads with two distinct peaks for STED (49.9 ± 2.3nm, n=28) vs. STED + DC (43.0 ± 2.7nm, n=29). **g,** Representative image of GATTA Dual Color 70±5nm spaced AlexaFluor-647/ AlexaFluor-594/ AlexaFluor-647 in confocal, STED, and STED + DC. Pixel size: 25nm. **h,** Average line plot profiles of AlexaFluor-647 (black line) with SEM (red shadow) and AlexaFluor-594 (black line) with SEM (green shadow) (confocal, n=30 beads; STED and STED+DC, n=33 beads). **i,** Average (p=0.0773, two-tailed Student's t-test, n = 33 beads) AlexaFluor-647/AlexaFluor-647 140nm spaced beads' peak-to-peak distance for STED (134 ± 2.8nm) vs. STED + DC (127 ± 2.4nm). **j,** Average (p=0.0768, two-tailed Student's t-test,

n = 33 beads) AlexaFluor-647/ AlexaFluor-594 70nm spaced beads' peak-to-peak distance for STED (67 $\pm$ 1.4nm) vs. STED + DC (63 $\pm$ 1.3nm). **k**, Average ($p<0.0001$, two tailed Student's t-test, n = 33 beads) FWHM of AlexaFluor-594 beads for STED (86 $\pm$ 2.2nm) vs. STED + DC (62 $\pm$ 1.9nm). Insets (**f, i, j**) represent example distance measurements. Dashed white lines (**d, g**) were drawn in the orientations as shown to obtain line plot profiles for quantification. Bars represent the mean $\pm$ SEM from a minimum of ten different regions. Scale bar (**a, d, g**): 0.2μm.

Supplementary Figure 3:

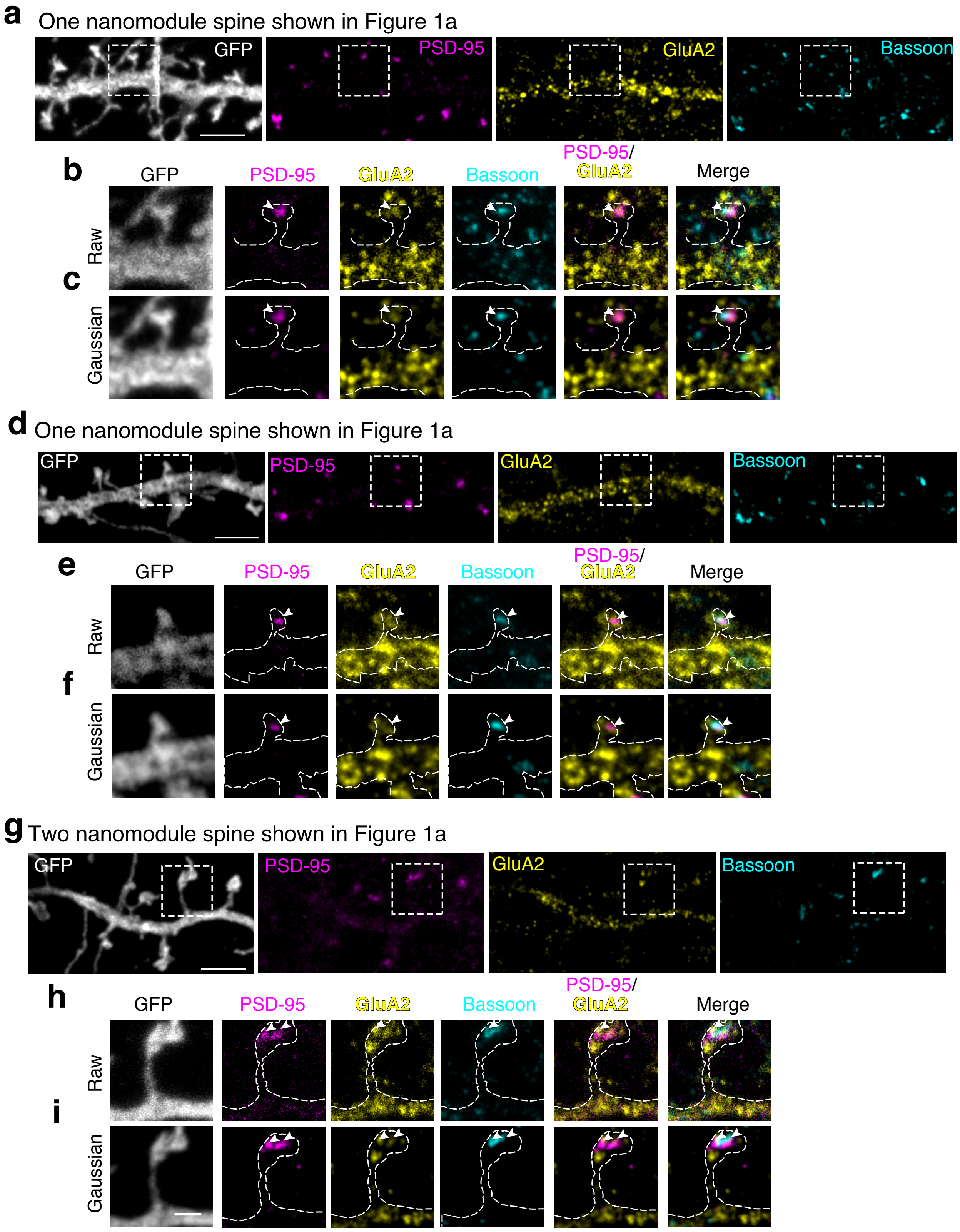

Supplementary Figure 3. Zoomed out and non-deconvolved GluA2 images of spines shown in Figure 1. Images are the example images in Figure 1 without deconvolution and with (**a, c, d, f, g, i**) or without Gaussian blur (**b**, **e**, **h**). The Gaussian size of two pixels was determined by the resolution of the system (see supplemental figure 2) so two pixel size gaussian blur better represents the actual analog sample being digitized by the STED scanner (see **b** vs **c**, **e** vs **f**, **h** vs **i**). **a**, **d**, **g** show the section dendrite containing example spines. Example spines are indicated by dashed box. **b, c, e, f**, **h**, **i** show example spines as in Figure 1, with (c, f, i) and without gaussian blur (b, e, h). PSD-95 (magenta), GluA2 (yellow) and Bassoon (cyan) were acquired in STED. GFP (gray) was acquired in the conventional confocal resolution. Scale bars (**a, d, g**): 3µm, (**i**): 1µm, applicable to images in **b, c, e, f, h, and i**.

Supplementary Figure 4:

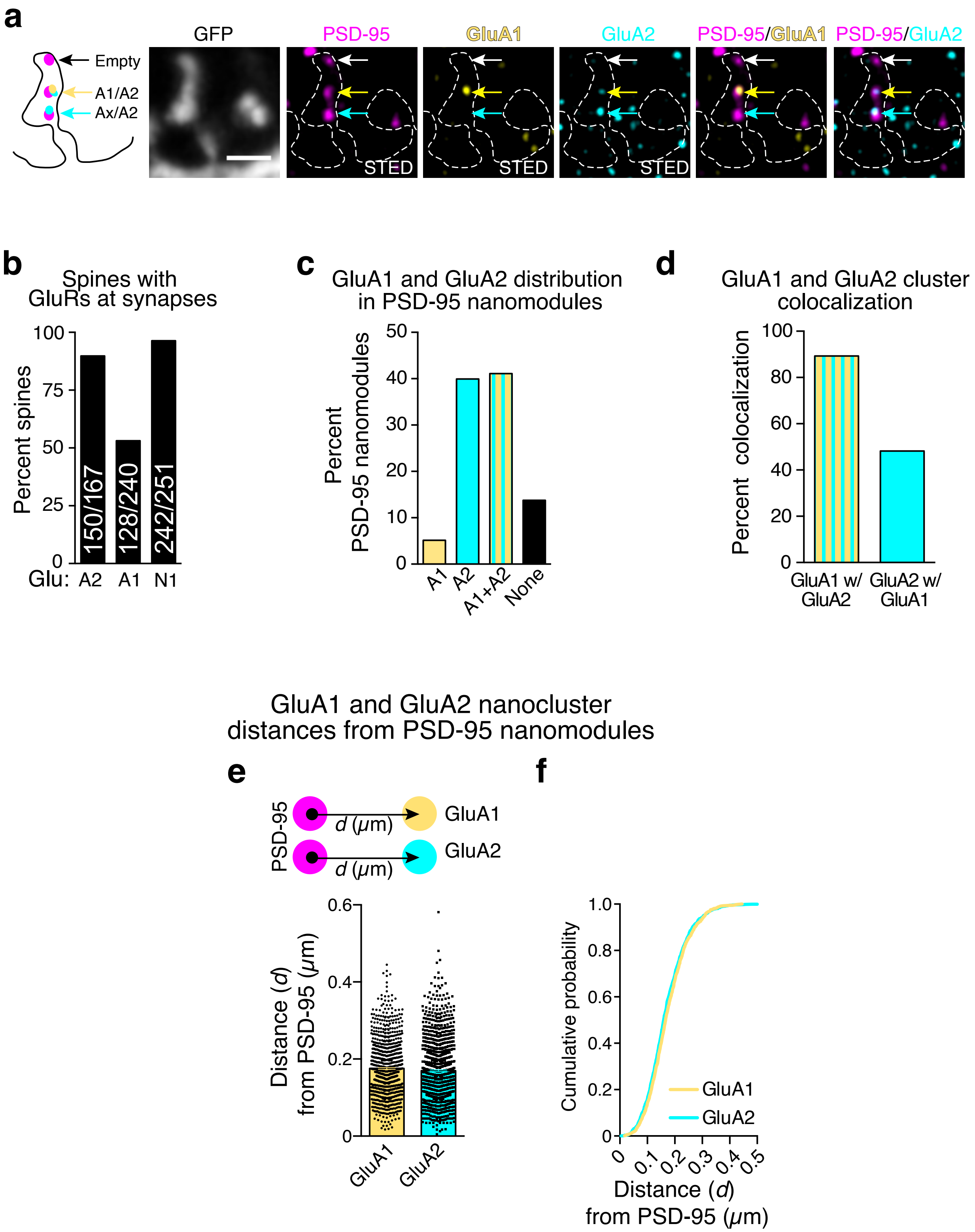

Supplementary Figure 4. GluA1 and GluA2-containing AMPARs are organized with different rules of modularity, yet their localization relative to PSD-95 is the same.

a, A three color STED image of a multi-nanomodule spine from a DIV21 cortical neuron transfected with GFP at DIV10 stained for endogenous PSD-95 (magenta), GluA1 (yellow), and GluA2 (cyan). The arrows identify PSD-95 nanomodules. The yellow arrow shows a single PSD-95 nanomodule with GluA1 and GluA2 subunits . Cyan arrow shows a PSD-95 nanomodule that containing only GluA2. The white arrow indicates a PSD-95 nanomodule without AMPARs. Scale bar: 1 µm. **b,** Percent of spines containing GluA2-containing AMPARs (A2) and GluA1-containing AMPARs (A1) or GluN1-containing NMDAR (N1). Numbers of spines analyzed and containing different subunit classes as indicated within each bar (containing/analyzed). **c,** The quantification of GluA1 and GluA2 AMPAR subunit distributions at PSD-95 nanomodules (n=428 nanoclusters). The majority of nanomodules contain either GluA1/GluA2 co-clusters (176/428 nanomodules, cyan/yellow bar) or GluA2 without GluA1 (171/428 nanomodules, cyan bar). A small fraction of PSD-95 nanomodules do not contain AMPARs (~14%, 59/428 nanomodules, black bar). GluA1 only containing PSD-95 nanomodules are rare (~5%, 22/428 nanomodules, yellow bar). **d,** Quantification of the colocalization of GluA1 and GluA2 clusters in PSD-95 nanomodules. Percent cluster colocalization was calculated for all GluA1-containing nanomodules (n=166, cyan/yellow bar) and for all GluA2-containing nanomodules (n=288, cyan bar). **e,** Average distances between the centers of PSD-95 nanomodules and either GluA1 (n= 940 PSD-95-colocalized GluA1 clusters) or GluA2 nanomodules (n=1472 PSD-95-colocalized GluA2

clusters, p=0.0643, two-tailed Student's t-test). **f,** Cumulative probability distributions of the distances between centers of PSD-95 and either GluA1 or GluA2 clusters (p=0.1458, two-tailed K-S test). Bars (**b, c, d**) represent means. Bars (**e**) represent the mean ± SEM.

Supplementary Figure 5:

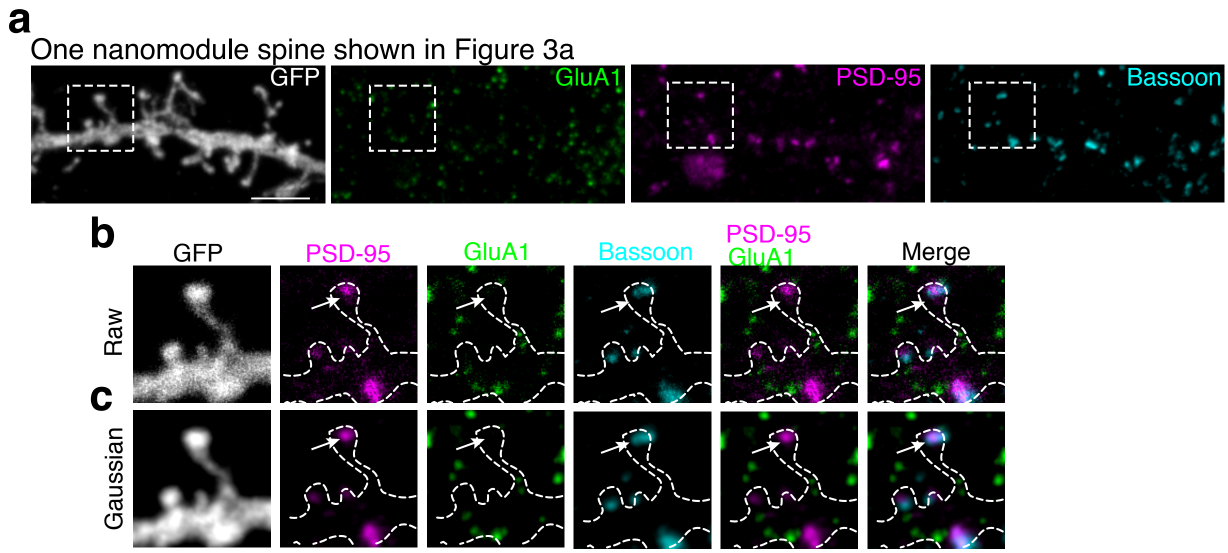

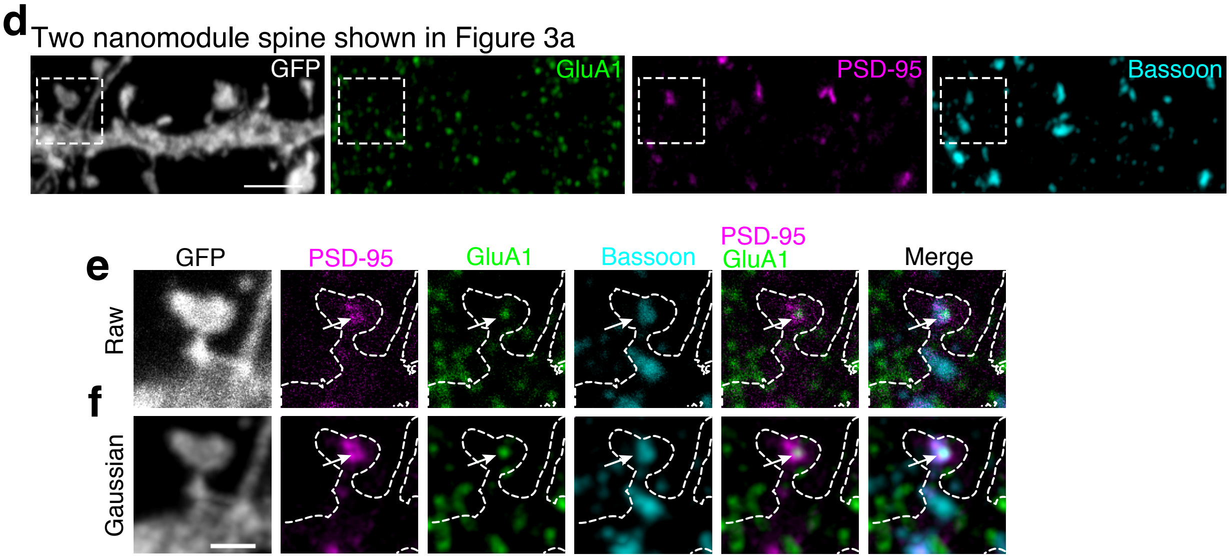

Supplementary Figure 5. Zoomed out and non-deconvolved GluA1 images of spines shown in Figure 3. Images are the example images in Figure 3 without deconvolution and with (**a, c, d, f**) or without Gaussian blur (**b**, **e**). The Gaussian size of two pixels was determined by the resolution of the system (see supplemental figure 2) so two pixel size gaussian blur better represents the actual analog sample being digitized by the STED scanner (see **b** vs **c**, **e** vs **f**). **a**, **d** show the section dendrite containing example spines. Example spines are indicated by dashed box. **b, c, e, f**, show example spines as in Figure 3, with (**c**, **f**) and without gaussian blur (**b**, **e**). PSD-95 (magenta), GluA1 (green) and Bassoon (cyan) were acquired in STED. GFP (gray) was acquired in the conventional confocal resolution. Scale bars (**a, d**): 3μm, (**f**): 1μm, applicable to images in **b, c, e,** and **f**.

Supplementary Figure 6:

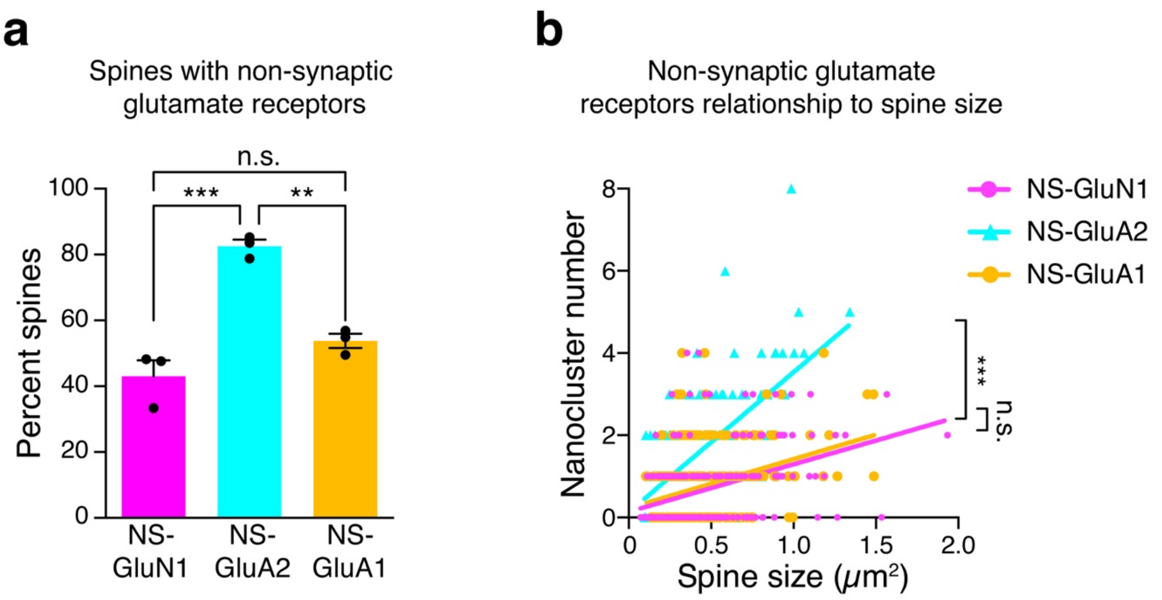

Supplementary Figure 6. Distribution of non-synaptic NMDAR and AMPAR nanoclusters in spines.

a, Percent of spines with at least one non-synaptic glutamate receptor nanocluster. Compared to spines containing non-synaptic GluN1 and GluA1 nanoclusters, significantly more spines contain non-synaptic GluA2 nanoclusters (***p=0.003, **p=0.0019, one-way ANOVA with Tukey's post-hoc, data were compiled from Figs. 1f, 2k and 3e). **b,** Relationship of the non-synaptic GluN1 (n = 251 spines), GluA2 (n=167 spines) and GluA1 (n=237 spines) nanocluster numbers to spine size. Non-synaptic GluA2 nanocluster numbers scale significantly better with spine size than either non-synaptic GluN1 or GluA1 (p<0.0001, one-way ANCOVA). Bars represent the mean ± SEM.

Supplementary Figure 7:

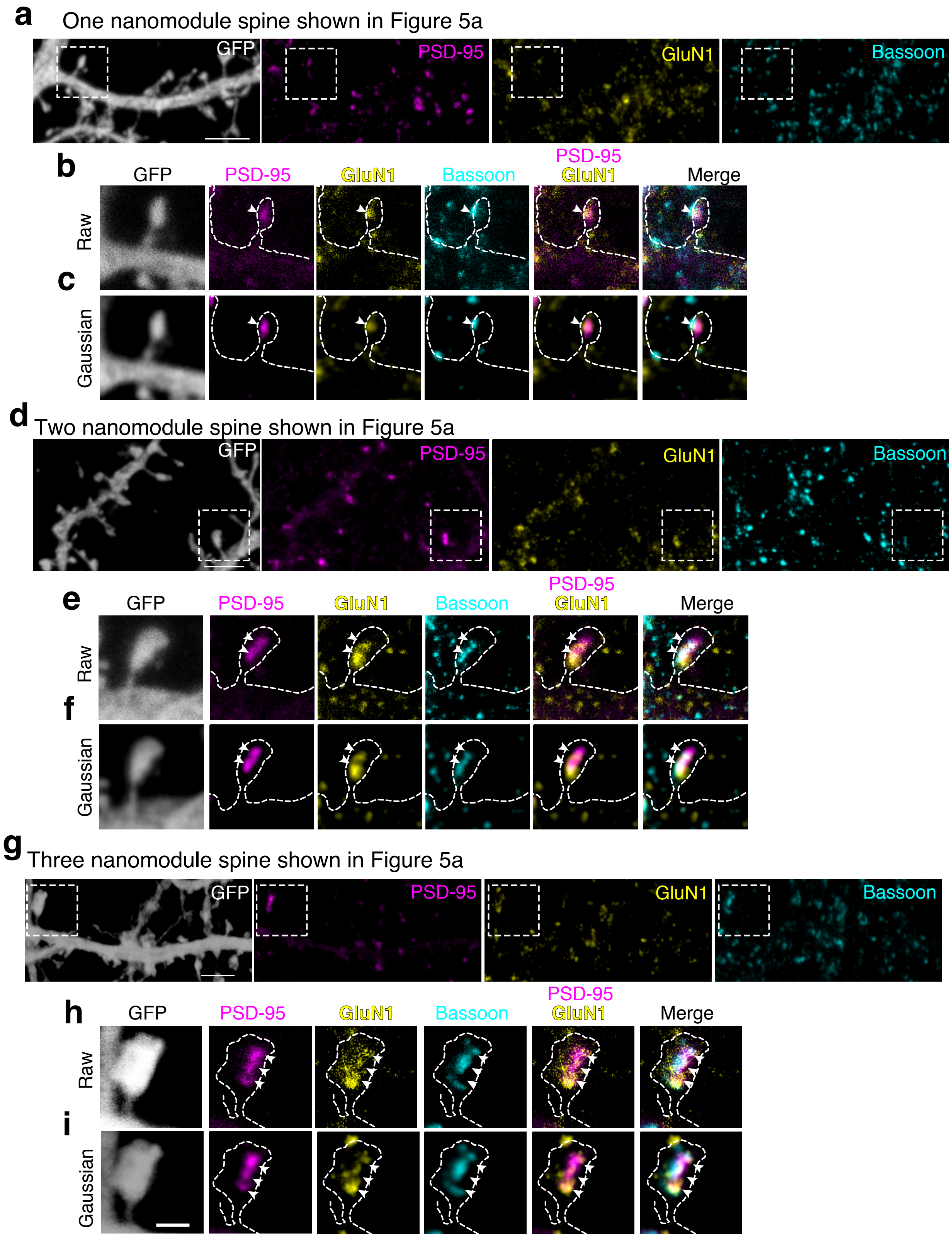

Supplementary Figure 7. Zoomed out and non-deconvolved GluN1 images of spines shown in Figure 5. Images are the example images in Figure 5 without deconvolution and with (**a, c, d, f, g, i**) or without Gaussian blur (**b**, **e**, **h**). The Gaussian size of two pixels was determined by the resolution of the system (see supplemental figure 2) so two pixel size gaussian blur better represents the actual analog sample being digitized by the STED scanner (see **b** vs **c**, **h** vs **i**). **a**, **d**, **g** show the section dendrite containing example spines. Example spines are indicated by dashed box. **b, c, e, f**, **h**, **i** show example spines as in Figure 5, with (**c**, **f**, **i**) and without gaussian blur (**b**, **e**, **h**). PSD-95 (magenta), GluN1 (yellow) and Bassoon (cyan) were acquired in STED. GFP (gray) was acquired in the conventional confocal resolution. Scale bars (**a, d, g**): 3μm, (**i**): 1μm, applicable to images in **b, c, e, f, h, and i**.

Supplementary Figure 8:

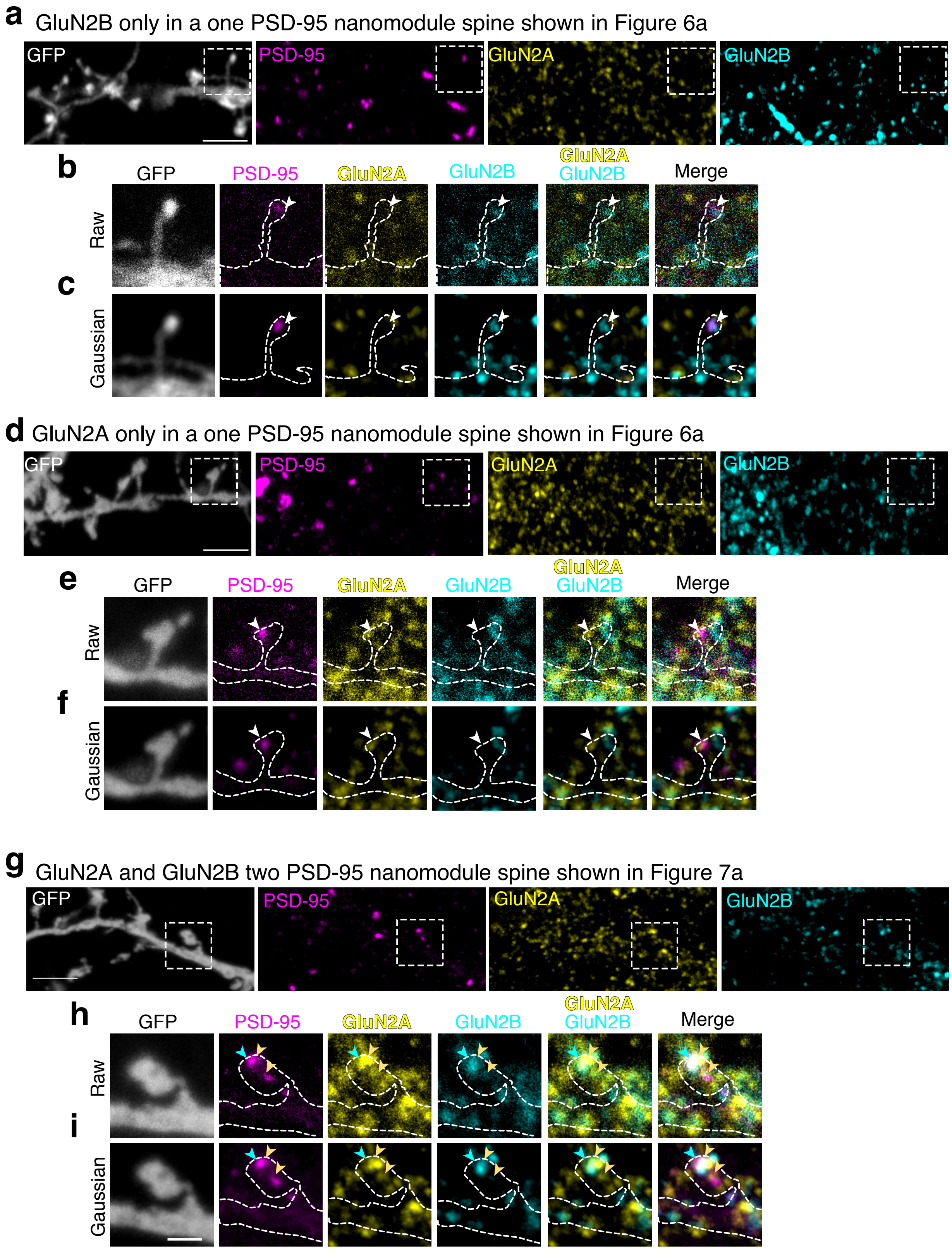

Supplementary Figure 8. Non-deconvolved GluN2A and GluN2B images related to Figures 6 and 7. Images are the example images in Figure 6 and 7 without deconvolution and with (**a, c, d, f, g, i**) or without Gaussian blur (**b**, **e**, **h**). The Gaussian size of two pixels was determined by the resolution of the system (see supplemental figure 2) so two pixel size gaussian blur better represents the actual analog sample being digitized by the STED scanner (see **b** vs **c**, **h** vs **i**). **a**, **d**, **g** show the section dendrite containing example spines. Example spines are indicated by dashed box. **b, c, e, f**, **h**, **i** show example spines as in Figure 6 and 7, with (**c**, **f**, **i**) and without gaussian blur (**b**, **e**, **h**). PSD-95 (magenta), GluN2A (yellow) and GluN2B (cyan) were acquired in STED. GFP (gray) was acquired in the conventional confocal resolution. Scale bars (**a, d, g**): 3μm, (**i**): 1μm, applicable to images in **b, c, e, f, h, and i**.z

Supplementary Figure 9:

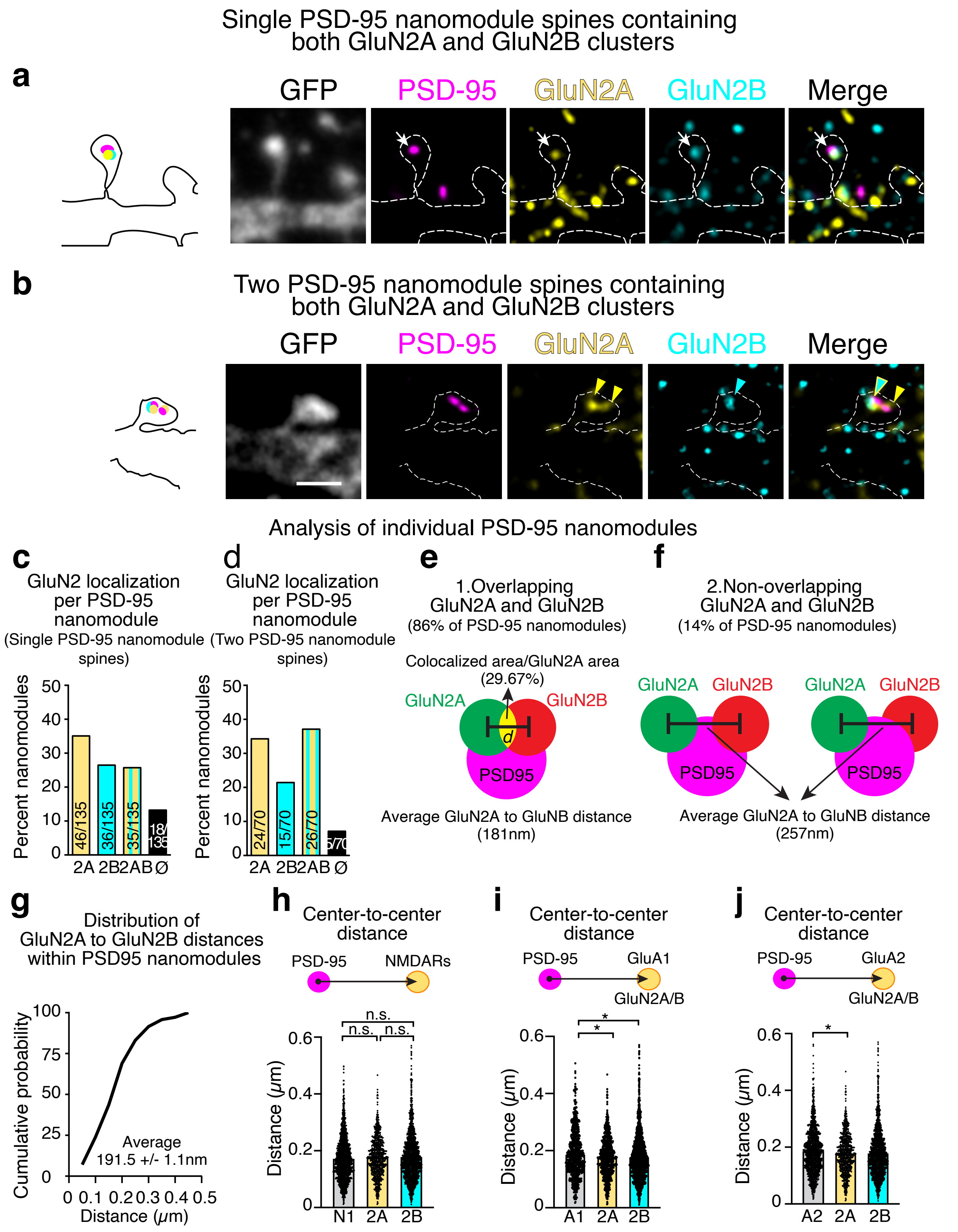

Supplementary Figure 9. NMDAR subtype distribution in single and two nanomodule spines.

a, A three-color STED image of single nanomodule containing spines from a DIV21 cortical neuron immunostained for PSD-95 (magenta), GluN2A (yellow), and GluN2B (cyan). The arrow indicates a PSD-95 nanomodule containing both GluN2A and GluN2B clusters. **b,** Three-color STED image of a two nanomodule spine from a DIV21 cortical neuron immunostained as in **a**. The yellow arrowheads indicate PSD-95 nanomodules containing GluN2A clusters; the cyan arrowhead indicates a PSD-95 nanomodule containing GluN2B. Scale bar (**b**): 1 μm, applicable to images in **a**. **c,** Percent of individual PSD-95 nanomodules containing the indicated GluN2 subtypes in all single nanomodule spines (n= 135 PSD-95 nanomodules). **d,** Percent of individual PSD-95 nanomodules containing the indicated GluN2 subtypes in all two nanomodule spines (n=70 PSD-95 nanomodules). **e,** Schematic demonstrating average distance and percent colocalization measurements calculated between overlapping GluN2A and GluN2B clusters colocalized with the same PSD-95 nanomodule (n= 61 PSD-95 nanomodules). **f,** Schematic demonstrating average distance measurements calculated between non-overlapping GluN2A and GluN2B clusters colocalized with the same PSD-95 nanomodule (n=10 PSD-95 nanomodules). **g,** Cumulative distribution of distances between centers of GluN2A and GluN2B nanoclusters colocalized with the same PSD-95 nanomodule (n=71 PSD-95 nanomodules) . **h,** Average ($p=0.0772$, one-way ANOVA with Tukey's post hoc) center-to-center distance measurements between PSD-95 and GluN1 (n= 1463 clusters), GluN2A (n= 746 clusters) and GluN2B (n= 1673 clusters). **i,** Average (**$p= 0.0072$, one-way ANOVA with Tukey's post hoc) center-to-center

distance measurements between PSD-95 and GluA1 (n=755 clusters), GluN2A (*p=0.0101) and GluN2B (*p=0.0213). **j,** Average (*p=0.0169, one-way ANOVA with Tukey's post hoc) center to center distance measurements between PSD-95 and GluA2 (n = 1457 clusters), GluN2A (*p = 0.0293) and GluN2B (p = 0.0624). Bars represent means.

Supplementary Figure 10:

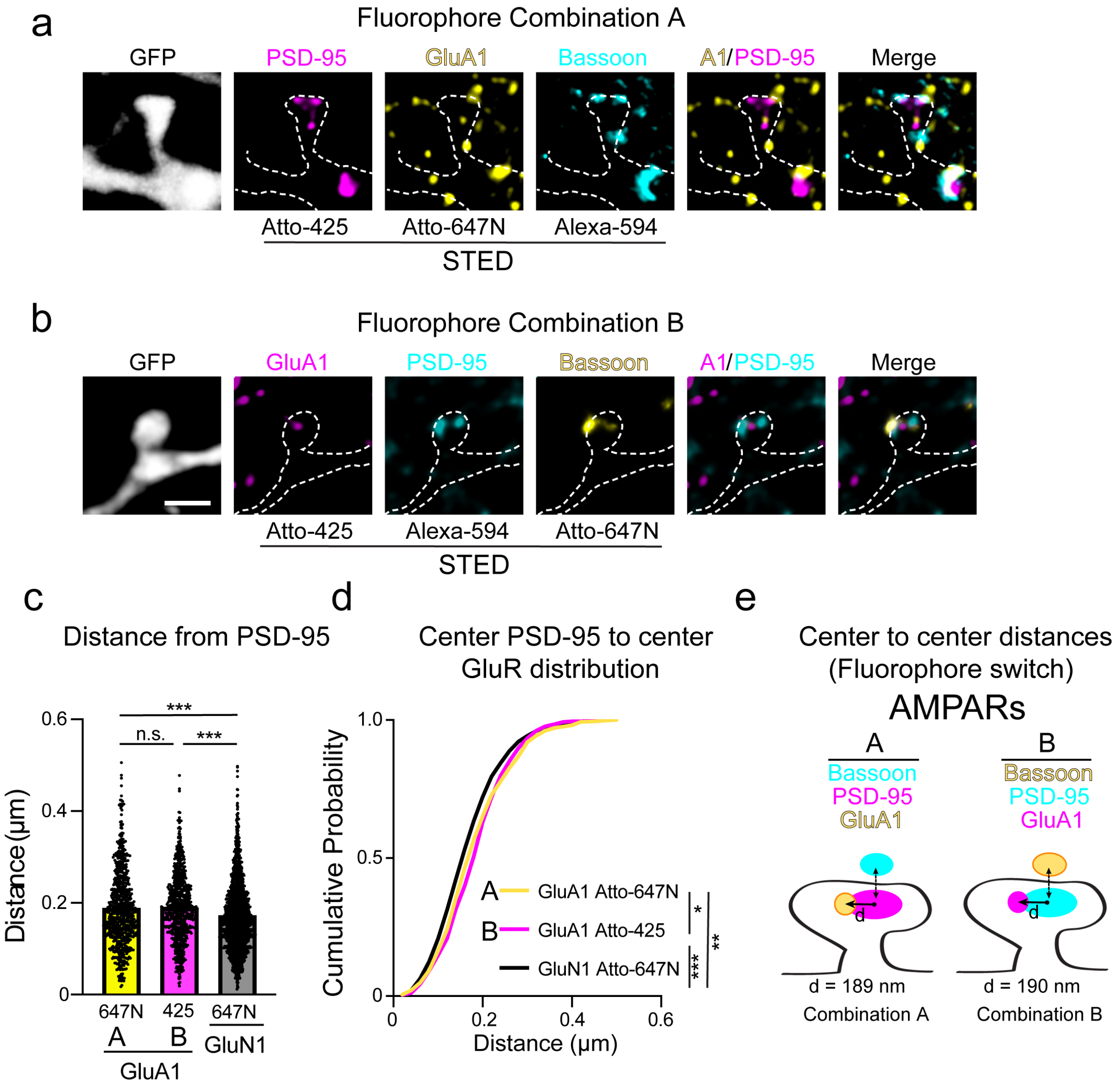

Supplementary Figure 10. GluA1 localization relative to PSD-95 is independent of secondary fluorophore labeling. a, b, Representative images of multi-nanomodule spines from DIV21 cortical neurons transfected with GFP at DIV10 and stained for endogenous PSD-95, GluA1 and Bassoon. Using the labeling combination A (**a**) neurons were stained for PSD-95 (magenta, Atto-425), GluA1 (yellow, Atto-647N) and Bassoon (cyan, AlexaFluor-594). In the labeling combination B (**b**) with swapped secondary antibodies, neurons were stained for GluA1 (magenta, Atto-425), PSD-95

(yellow, AlexaFluor-594) and Bassoon (cyan, Atto-647N). Scale bar (b): 1 μm, applicable to images in **a**. **c,** Averages (****p<0.0001, one-way ANOVA with Tukey's post-hoc) and (**d**) cumulative probability distributions (GluA1-Atto-647N vs. GluA1-Atto-425 *p=0.0144, GluA1-Atto-647N vs. GluN1-Atto-647N **p=0.0023, GluA1-Atto-425 vs. GluN1-Atto-647N ****p<0.0001, two-tailed K-S test) of center-to-center distances between PSD-95 and co-localized GluA1-Atto-647N (yellow, n=755 clusters), GluA1-Atto-425 (magenta, n=704 clusters), and NMDARs (GluN1, grey, n=1463 clusters). **e,** Schematic of Combination A (left) and B (right) labeling schemes indicating measurement approach and average center-to-center distances between PSD-95 and co-localized GluA1. Measurements in (**c)** and (**d**) were performed on a per cluster basis (see Methods). Bars represent the mean ± SEM.

Supplementary Figure 11:

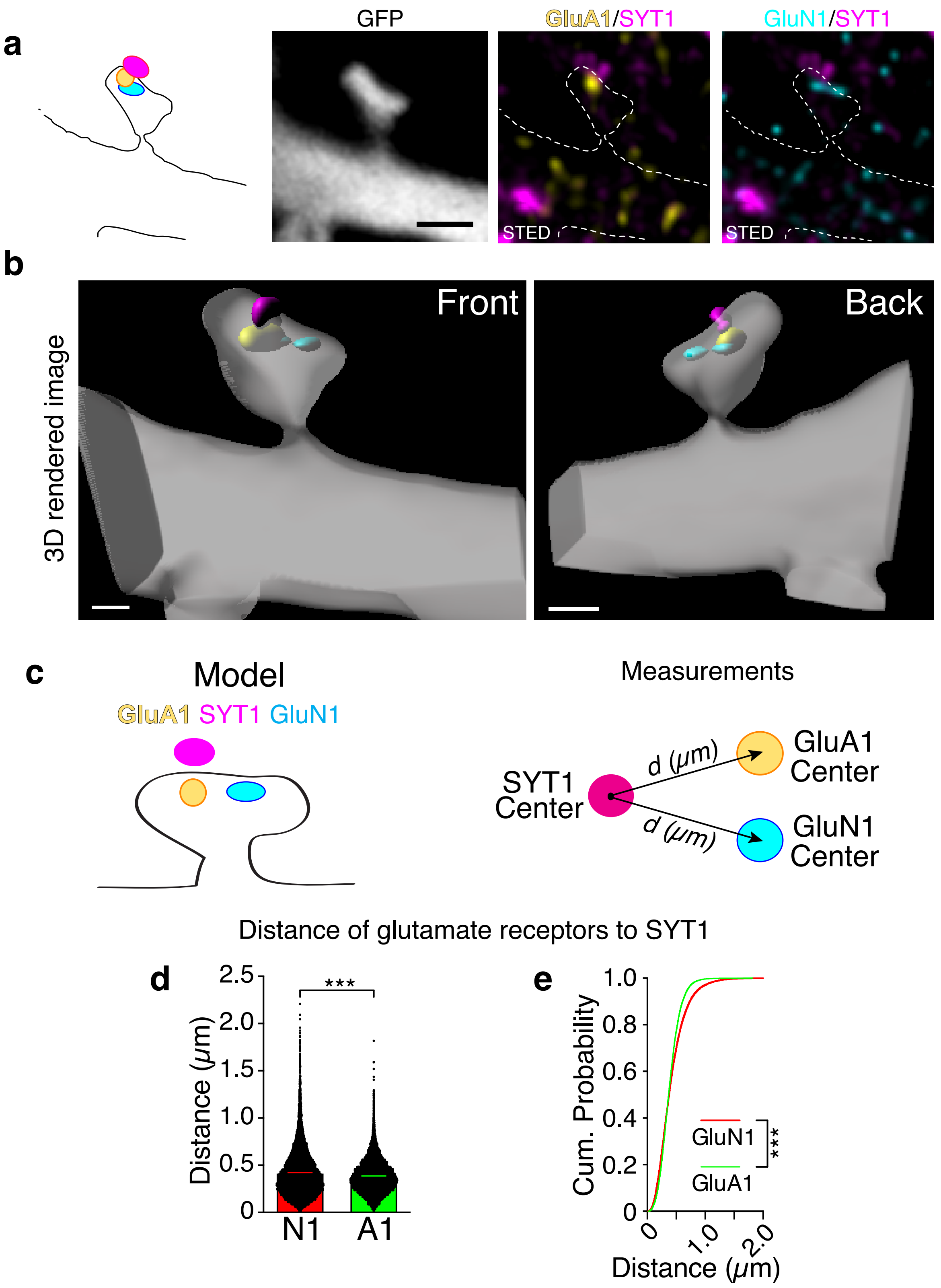

Supplementary Figure 11. The SYT1 calcium sensor is organized closer to AMPAR nanomodules than NMDAR nanomodules.

a, Image of a GFP-labeled spine (gray, dotted outline, confocal mode) containing colocalized GluA1 (yellow) and GluN1 (cyan) nanoclusters juxtaposed to a single SYT1 cluster (magenta). Scale bar: 1 µm. **b,** Three-dimensional reconstruction using Imaris software. Example of the closer apposition between GluA1 and SYT1 than between GluN1 and SYT1 is shown. Scale bars, Front = 300 nm, Back = 500 nm. **c,** A model of the trans-synaptic organization between AMPAR, NMDAR, and SYT1 nanomodules in a single spine. The organization of SYT1 relative to AMPAR (GluA1) and NMDAR (GluN1) nanoclusters was determined by measuring the distances between the centers of SYT1 and glutamate receptor subunits as depicted. **d,** Average distances between the centers of SYT1 nanomodules and either GluA1 (n=13958 clusters) or GluN1 (n=14181 clusters, ***p<0.0001, two-tailed Student's t-test). **e,** Cumulative probability distributions of distances between the centers of SYT1 and either GluA1 or GluA2 clusters (***p<0.0001, two-tailed K-S test). Bars represent the mean ± SEM.
