# Supplementary material for: Nanoscale rules governing the organization of glutamate receptors in spine synapses are subunit specific
Source: Nat Commun. 2022 Feb 17;13:920. doi: 10.1038/s41467-022-28504-4 (PMC8854560; doi:10.1038/s41467-022-28504-4)
Supplement: Supplementary file 2 — Reporting Summary [file 41467_2022_28504_MOESM2_ESM.pdf]

nature research

Corresponding author(s): Matthew Dalva

Last updated by author(s): Jan 21, 2021

Reporting Summary

Nature Research wishes to improve the reproducibility of the work that we publish. This form provides structure for consistency and transparency in reporting. For further information on Nature Research policies, see our Editorial Policies and the Editorial Policy Checklist.

Statistics

For all statistical analyses, confirm that the following items are present in the figure legend, table legend, main text, or Methods section.

n/a	Confirmed	
☐	☒	The exact sample size (*n*) for each experimental group/condition, given as a discrete number and unit of measurement
☐	☒	A statement on whether measurements were taken from distinct samples or whether the same sample was measured repeatedly
☐	☒	The statistical test(s) used AND whether they are one- or two-sided<br>*Only common tests should be described solely by name; describe more complex techniques in the Methods section.*
☐	☒	A description of all covariates tested
☐	☒	A description of any assumptions or corrections, such as tests of normality and adjustment for multiple comparisons
☐	☒	A full description of the statistical parameters including central tendency (e.g. means) or other basic estimates (e.g. regression coefficient) AND variation (e.g. standard deviation) or associated estimates of uncertainty (e.g. confidence intervals)
☐	☒	For null hypothesis testing, the test statistic (e.g. *F*, *t*, *r*) with confidence intervals, effect sizes, degrees of freedom and *P* value noted<br>*Give P values as exact values whenever suitable.*
☒	☐	For Bayesian analysis, information on the choice of priors and Markov chain Monte Carlo settings
☒	☐	For hierarchical and complex designs, identification of the appropriate level for tests and full reporting of outcomes
☐	☒	Estimates of effect sizes (e.g. Cohen's *d*, Pearson's *r*), indicating how they were calculated

Our web collection on statistics for biologists contains articles on many of the points above.

Software and code

Policy information about availability of computer code

Data collection	STED images were collected using a Leica TCS SP8 gated STED (gSTED) 3X superresolution system (Leica Microsystems) equipped with a tunable white light laser, CW 592 nm and 660 nm depletion lines, and a pulsed 775 nm depletion line. Live cell images were collected with either Leica SP8 or confocal spinning disk with a Yokogawa CSU-10 and a Hamamatsu EM-CCD digital camera attached to an inverted Leica microscope and controlled by Metamorph software 7.10.
Data analysis	No programs were written for this manuscript. Image analysis was conducted off-line using Fiji Image J (https://imagej.net/Fiji) and built-in macros. 3D rendering of STED images was performed using the Imaris software (Bitplane AG 8.3.1). Statistics was performed using Graphpad Prism 8.0. Power calculations were performed using G*Power software 3.1.

For manuscripts utilizing custom algorithms or software that are central to the research but not yet described in published literature, software must be made available to editors and reviewers. We strongly encourage code deposition in a community repository (e.g. GitHub). See the Nature Research guidelines for submitting code & software for further information.

Data

Policy information about availability of data

All manuscripts must include a data availability statement. This statement should provide the following information, where applicable:

- Accession codes, unique identifiers, or web links for publicly available datasets
- A list of figures that have associated raw data
- A description of any restrictions on data availability

The data that support the findings of this study are available from the corresponding author upon reasonable request. There are no restriction on data availability. The data availability statement is included with the manuscript.

Field-specific reporting

Please select the one below that is the best fit for your research. If you are not sure, read the appropriate sections before making your selection.

☒ Life sciences ☐ Behavioural & social sciences ☐ Ecological, evolutionary & environmental sciences

For a reference copy of the document with all sections, see nature.com/documents/nr-reporting-summary-flat.pdf

Life sciences study design

All studies must disclose on these points even when the disclosure is negative.

Sample size	Standard power calculations using the power of 0.8-0.95 and alpha = 0.05 were used to determine the sample size. Power calculations were performed in the G*power 3.1 software using tests and variance based on our previously published data (Hruska et al., 2015, Hruska et al., 2018, Dalva et al., 2000, Kayser et al., 2008, McClelland et al., 2010 and Nolt et al., 2011).
Data exclusions	No data were excluded from our analyses.
Replication	Each experiment was replicated a minimum of three times and data was reliably reproduced with each replication attempt.
Randomization	Data were acquired and analyzed based on the standards in the field and our previously published results (Hruska et al., 2015, Hruska et al., 2018, Dalva et al., 2000, Kayser et al., 2008, McClelland et al., 2010 and Nolt et al., 2011). However, no method of randomization was used to determine how samples were allocated to experimental groups and processed since the experiments were done entirely in vitro. Because no animals were used in ayn experimental groups, randomization was not relevant.
Blinding	Data for spine analysis of module number represent observations and were acquired and analyzed with an experimenter blinded to the condition. Experimenter as well as analyzer were blinded to conditions.

Reporting for specific materials, systems and methods

We require information from authors about some types of materials, experimental systems and methods used in many studies. Here, indicate whether each material, system or method listed is relevant to your study. If you are not sure if a list item applies to your research, read the appropriate section before selecting a response.

Materials & experimental systems

n/a	Involved in the study	
☐	☒	Antibodies
☒	☐	Eukaryotic cell lines
☒	☐	Palaeontology and archaeology
☐	☒	Animals and other organisms
☒	☐	Human research participants
☒	☐	Clinical data
☒	☐	Dual use research of concern

Methods

n/a	Involved in the study	
☒	☐	ChIP-seq
☒	☐	Flow cytometry
☒	☐	MRI-based neuroimaging

Antibodies

Antibodies used

Primary antibodies:
mouse monoclonal (IgG2A) anti-PSD-95 clone K28/43(1:200, Neuromab, UC Davis, Davis, CA),
mouse monoclonal IgG1 anti-PSD-95 clone 7E3-1B8 (1:250, Thermo Fisher Scientific, Waltham, MA, cat#: MA1-046)
anti-guinea pig polyclonal anti-Bassoon (1:300, Synaptic Systems, Gottingen, Germany, cat# 141004),
rabbit polyclonal anti-Bassoon (1:300, Synaptic Systems, cat #: 141 003),
chicken anti-GFP (1:2000, Abcam, Cambridge, MA, cat# ab13970),
rabbit monoclonal anti-GluN1 (1:500, AB9864, Millipore Sigma),
mouse monoclonal (IgG2A) anti-GluN2A clone N327/95 (1:250, Neuromab),
mouse monoclonal (IgG2B) anti-GluN2B clone N59/3625(1:250, Neuromab),
mouse monoclonal (IgG1) anti-GluA1 clone N355/11 (1:250, Neuromab),
rabbit polyclonal anti-GluA2 (1:500, Synaptic Systems, cat#: 182 103),
mouse monoclonal (IgG2A) anti-Synaptotagmin 1 (1:500, Synaptic Systems, cat#: 105011);

Secondary antibodies:
Goat anti mouse IgG2A Atto 425 (1:250, Rockland, Inc.,cat# 610-151-041),
Goat anti-mouse IgG1 Atto-425 (1:250, Rockland, cat#: 610-151- 040),
Goat anti-rabbit Atto 425 (1:250, Rockland, cat#: 611-151-122),

	Goat anti-mouse IgG1 Atto-647N (1:500, Rockland, Inc., cat # 610-156-040),<br>Goat anti-mouse IgG2A Atto-647N (1:500, Rockland, cat#: 610-156-041),<br>Goat anti mouse IgG2B Atto-647N (1:500, Rockland, cat#: 610-156-042),<br>Goat anti-rabbit Atto-647N (1:500, Rockland, Inc., cat # 611-156-122),<br>Goat anti-mouse IgG1 AlexaFluor-594 (1:500, Jackson ImmunoResearch, cat# 115-587-185),<br>Goat anti-mouse IgG2A AlexaFlour-594 (1:500,Jackson ImmunoResearch, cat# 115-585-206),<br>Donkey anti guinea pig AlexaFluor-594 (1:500, Jackson ImmunoResearch, cat # 706-586-148)<br>Donkey anti-rabbit AlexaFluor-594 (1:500, Jackson ImmunoResearch, cat # 711-585-152).
Validation	All primary antibodies were validated in the KO animals by the respective vendors. Additionally, all primary antibodies were profiled in previous publications and were reported to be specific:<br><br>mouse monoclonal (IgG2A) anti-PSD-95 clone K28/43(1:200, Neuromab, UC Davis, Davis, CA) (Hruska et al., 2015; Hruska et al., 2018)<br>mouse monoclonal IgG1 anti-PSD-95 clone 7E3-1B8 (1:250, Thermo Fisher Scientific, Waltham, MA, cat#: MA1-046) (Schaffer et al., 2018; Abrahamsson et al., 2017)<br>anti-guinea pig polyclonal anti-Bassoon (1:300, Synaptic Systems, Gottingen, Germany, cat# 141004) (Hruska et al., 2018; Schroeder et al., 2021)<br>rabbit polyclonal anti-Bassoon (1:300, Synaptic Systems, cat #: 141 003) (Hruska et al., 2018; Nishida et al., 2021)<br>chicken anti-GFP (1:2000, Abcam, Cambridge, MA, cat# ab13970) (Greer et al., 2021; Steevens et al., 2021)<br>rabbit monoclonal anti-GluN1 (1:500, AB9864, Millipore Sigma) (Gleichman et al., 2012; Peng et al., 2014)<br>mouse monoclonal (IgG2A) anti-GluN2A clone N327/95 (1:250, Neuromab) (Balsara et al., 2015)<br>mouse monoclonal (IgG2B) anti-GluN2B clone N59/3625(1:250, Neuromab) (Washburn et al., 2020; Hruska et al., 2015)<br>mouse monoclonal (IgG1) anti-GluA1 clone N355/11 (1:250, Neuromab) (Moraga-Amaro et al., 2016)<br>rabbit polyclonal anti-GluA2 (1:500, Synaptic Systems, cat#: 182 103) (Won et al., 2019; Fiuza et al., 2017)<br>mouse monoclonal (IgG2A) anti-Synaptotagmin 1 (1:500, Synaptic Systems, cat#: 105011) (Farsi et al., 2021; Hoffmann-Conaway et al., 2020)

Animals and other organisms

Policy information about studies involving animals; ARRIVE guidelines recommended for reporting animal research

Laboratory animals	Long-Evans E17-18 rat embryos from timed pregnant animals purchased from Charles River Laboratories Inc. (Wilmington, MA) were used to make primary cortical neuron cultures. Both male and female embryos were used.
Wild animals	The study did not involve the use of wild animals.
Field-collected samples	The study did not involve samples collected from the field.
Ethics oversight	All animal studies were approved by the Institutional Animal Care and Use Committee guidelines at Thomas Jefferson University in accordance with the US National Institutes of Health guidelines.

Note that full information on the approval of the study protocol must also be provided in the manuscript.
